# Immunomodulatory Microparticles Epigenetically Modulate T Cells and Systemically Ameliorate Autoimmune Arthritis

**DOI:** 10.1002/advs.202202720

**Published:** 2023-03-08

**Authors:** David A. McBride, Matthew D. Kerr, Wade T. Johnson, Anders Nguyen, Martina Zoccheddu, Mina Yao, Edward B. Prideaux, Nicholas C. Dorn, Wei Wang, Mattias N.D. Svensson, Nunzio Bottini, Nisarg J. Shah

**Affiliations:** ^1^ Department of Nanoengineering University of California La Jolla San Diego CA 92093 USA; ^2^ Chemical Engineering Program University of California La Jolla San Diego CA 92093 USA; ^3^ Department of Rheumatology and Inflammation Research Sahlgrenska Academy Institute of Medicine University of Gothenburg Gothenburg 41346 Sweden; ^4^ Department of Medicine Division of Rheumatology Allergy and Immunology University of California La Jolla San Diego CA 92093 USA; ^5^ Department of Chemistry and Biochemistry University of California La Jolla San Diego CA 92093 USA; ^6^ Department of Cellular and Molecular Medicine University of California La Jolla San Diego CA 92093 USA

**Keywords:** immune engineering, regulatory T cells, rheumatoid arthritis

## Abstract

Disease modifying antirheumatic drugs (DMARDs) have improved the prognosis of autoimmune inflammatory arthritides but a large fraction of patients display partial or nonresponsiveness to front‐line DMARDs. Here, an immunoregulatory approach based on sustained joint‐localized release of all‐trans retinoic acid (ATRA), which modulates local immune activation and enhances disease‐protective T cells and leads to systemic disease control is reported. ATRA imprints a unique chromatin landscape in T cells, which is associated with an enhancement in the differentiation of naïve T cells into anti‐inflammatory regulatory T cells (T_reg_) and suppression of T_reg_ destabilization. Sustained release poly‐(lactic‐*co*‐glycolic) acid (PLGA)‐based biodegradable microparticles encapsulating ATRA (PLGA‐ATRA MP) are retained in arthritic mouse joints after intra‐articular (IA) injection. IA PLGA‐ATRA MP enhance migratory T_reg_ which in turn reduce inflammation and modify disease in injected and uninjected joints, a phenotype that is also reproduced by IA injection of T_reg_. PLGA‐ATRA MP reduce proteoglycan loss and bone erosions in the SKG and collagen‐induced arthritis mouse models of autoimmune arthritis. Strikingly, systemic disease modulation by PLGA‐ATRA MP is not associated with generalized immune suppression. PLGA‐ATRA MP have the potential to be developed as a disease modifying agent for autoimmune arthritis.

## Graphical abstract

This work reports a new intraarticular drug delivery strategy of a disease‐modifying agent that can promote durable disease remission in autoimmune arthritis. The agent protects joints from inflammation‐mediated damage while avoiding generalized suppression of immunity. The systemic effect is attributed to epigenetic modulation of T cells by the agent, which enhances and stabilizes disease‐protective regulatory T cells (T_reg_). [Image composed in part using BioRender.]

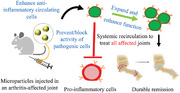

## 1 Introduction

Inflammatory arthritides such as rheumatoid arthritis (RA) are systemic autoimmune inflammatory joint disorders that lead to chronic pain and disability. Disease modifying anti‐rheumatic drugs (DMARDs) that target cytokine signaling have greatly improved the prognosis of RA. However, a large fraction of patients display intolerance, partial or nonresponsiveness toward frontline DMARDs, and struggle to achieve disease control.^[^ ^1^ ^]^ Moreover, DMARDs operate through generalized immunosuppression and increase the iatrogenic risk of opportunistic and serious infections and some DMARDs could impair immune responses to vaccines.^[^ ^2^, ^3^, ^4^ ^]^ There is an unmet need for DMARDs that operate through immunoregulation to address pathogenic joint inflammation, rather than immunosuppression, for enhancing RA control in patients.

An emerging observation is that inflammatory changes in one RA‐affected joint can enhance inflammation in other joints through systemic cell recirculation. For example, immune cells with unique transcriptomic profiles have been shown to circulate systemically and contribute to synovitis.^[^ ^5^, ^6^, ^7^, ^8^, ^9^, ^10^, ^11^, ^12^, ^13^, ^14^ ^]^ Importantly, T cells can migrate from the bloodstream to the synovial tissue where they play a key role in inducing local RA joint inflammation.^[^ ^15^, ^16^, ^17^, ^18^ ^]^ RA‐affected joints are infiltrated with hyperactivated immune cells, including pathogenic CD4^+^ T cells which produce proinflammatory mediators and have specificity to joint autoantigens.^[^ ^15^ ^]^ Such CD4^+^ T cells comprise pathogenic T helper (Th) cells, which are proinflammatory, and disease protective T cells expressing FoxP3, called regulatory T cells (T_reg_). T_reg_ generally suppress inflammatory T cells but a numerical imbalance between T_reg_ and autoreactive proinflammatory CD4^+^ T cell subsets, including retinoic acid receptor‐related orphan receptor gamma (ROR*γ*)t‐expressing Th17 cells, and impaired T_reg_ function in chronically inflamed joints and draining lymph nodes are major contributors to the pathogenesis of RA. In response to pathogenic inflammation, T_reg_ may lose immunoregulatory function and convert to an “ex‐T_reg_” phenotype which produces proinflammatory cytokines, such as interleukin (IL)‐17.^[^ ^19^, ^20^, ^21^ ^]^

Here, we describe and validate an approach based on microparticles (MP) delivered via an intra‐articular (IA) injection which locally release an immunomodulatory agent that causes local enhancement of T_reg_ through epigenetic modulation. Since pathogenic T cells as well as protective T_reg_ are known to systemically recirculate throughout immune organs, our hypothesis is that locally enhanced T_reg_, via systemic recirculation, can systemically suppress joint‐specific autoimmunity without harming protective immune responses. The MP are composed of all‐trans retinoic acid (ATRA) encapsulated in hydrolytically degradable poly‐(lactic‐co‐glycolic) acid (PLGA) to generate PLGA‐ATRA MP that enables joint‐localized, sustained release of ATRA. Using the SKG mouse model of RA, we show that ATRA enhances differentiation of naïve mouse into T_reg_, an effect that was recapitulated with human T cells. ATRA persistently stabilizes SKG T_reg_ in conditions that otherwise favor trans‐differentiation into inflammatory Th17 cells. ATRA increases chromatin accessibility at T_reg_‐associated loci and decreases accessibility at Th17‐associated loci in a manner that coincides with differences in H3K4me3 histone methylation at the *Foxp3* locus and T_reg_ function‐associated loci. In SKG mice with established arthritis, PLGA‐ATRA MP reside in an arthritic joint for several weeks after a single IA injection. Bioactive ATRA released from IA PLGA‐ATRA MP reduces inflammation and enhances immunoregulatory cells including T_reg_ in the injected joint, which correlates with reduced inflammatory markers, bone erosions, and cartilage proteoglycan (PG) loss scores in both injected and uninjected joints without evidence of nonspecific suppression of T cell‐dependent immune responses. The immunomodulatory effect of IA PLGA‐ATRA MP was validated in the collagen‐induced arthritis (CIA) mouse model where similar reduction in arthritis severity in injected and uninjected joints with protection against bone erosions and cartilage PG loss was quantified.

## 2 Results

### 2.1 ATRA Promotes T_reg_ Differentiation and Stability in Mouse and Human T Cells Ex Vivo

**Figure 1:**
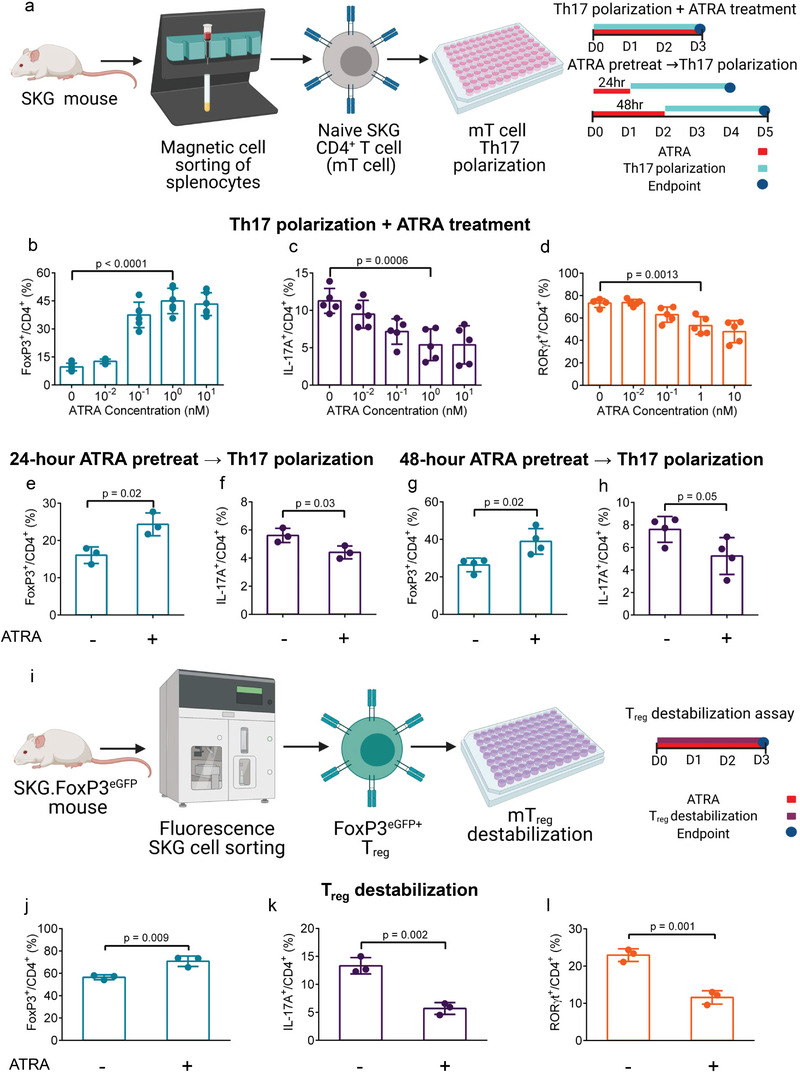
All‐trans retinoic acid (ATRA) differentially promotes T_reg_ enhancement and Th17 suppression in a concentration dependent manner. a) Experimental schematic and timeline for assessing the effect of ATRA on Th17 and T_reg_ differentiation from naïve CD4^+^ SKG T (mT) cells. b–d) Quantification of b) FoxP3, c) IL‐17A, and d) ROR*γ*t expression in CD4^+^ mT cells differentiated in Th17 inducing conditions with ATRA added at the depicted concentrations. e–h) Quantification of FoxP3 and IL‐17A expression in CD4^+^ T cells pretreated with or without 1 × 10^−9^ m ATRA for either e,f) 24 or g,h) 48 h prior to Th17 induction for an additional 72 h. i) Experimental schematic for assessing SKG T_reg_ (mT_reg_) destabilization. j–l) Quantification of j) FoxP3 expression, k) IL‐17A expression, and l) ROR*γ*t expression in T_reg_ following an IL‐6 mediated destabilization assay with or without 1 × 10^−9^ m ATRA. Data in (b)–(h) and (j)–(l) are the means ± SD of technical replicates from representative experiments; Data in (b)–(d), (j)–(l), and (e)–(h) are representative of three experimental replicates. Statistical analyses in (b)–(d) were performed using one‐way ANOVA with post hoc Dunnett's test; statistical analyses in (e)–(h) and (j)–(l) were performed using unpaired Student's two tailed *t*‐tests. Schematics in (a) and (i) were composed using BioRender.

To test the effect of ATRA on T cells differentiation, an ex vivo differentiation and stabilization assay in Th17 inflammatory conditions using cytokine supplementation was used (**Figure** **1**). Naïve SKG CD4^+^ T cells were isolated, which were consistently greater than 80% CD4^+^CD44^−^CD62L^+^ post‐enrichment (Figure S1a, Supporting Information). Subsequently, these cells were stimulated with anti‐mouse CD3 (*α*CD3) and anti‐mouse CD28 (*α*CD28) antibodies along with Th17 polarizing cytokines IL‐6, TGF‐*β*1, IL‐1*β*, and IL‐23, and ATRA was added to the T cell culture medium, with concentrations ranging from 10 × 10^−12^ to 10 × 10^−9^ m. Immunophenotypic analysis after 4 days showed that ATRA differentially enhanced FoxP3 and suppressed IL‐17A expression in a concentration‐dependent manner (Figure S1b, Supporting Information). At a concentration greater than 100 × 10^−12^ m, ATRA consistently enhanced FoxP3 expression (40.0 ± 3.4%) while below 100 × 10^−12^ m, 11.2 ± 2.3% of T cells expressed FoxP3 (Figure 1b). The fraction of IL‐17A^+^CD4^+^ T cells was comparable at 10 × 10^−12^ m ATRA relative to control (9.5 ± 1.7% vs 11.3 ± 1.5%). ATRA reduced the expression of IL‐17A at concentrations of 100 × 10^−12^ m (7.2 ± 1.5%), 1 × 10^−9^ m (5.4 ± 1.9%), and 10 × 10^−9^ m (5.4 ± 2.3%) (Figure 1c). In addition to reduced expression of IL‐17A, ATRA comparably reduced the fraction of ROR*γ*t^+^CD4^+^ T cells at 1 × 10^−9^ m (53.3 ± 7.9%) and 10 × 10^−9^ m (47.9 ± 9.9%) relative to cells not exposed to ATRA (73.3 ± 3.9%) (Figure 1d).

To test the persistence of the effect of ATRA‐mediated T_reg_ enhancement, naïve CD4^+^ T cells were stimulated with *α*CD3‐ and *α*CD28‐coated Dynabeads and 1 × 10^−9^ m ATRA was added for 24 or 48 h. Subsequently, the cells were thoroughly washed and replated in Th17 polarizing conditions for an additional 72 h without ATRA (Figure 1a). Pretreatment of naïve CD4^+^ T cells with ATRA for 24 h was sufficient to induce T_reg_ differentiation and inhibit Th17 differentiation (Figure 1e,f). The fraction of naïve CD4^+^ T cells that differentiated into T_reg_ increased further when the cells were pretreated with ATRA for 48 h, compared to untreated cells (Figure 1g,h). The fraction of IL‐17A^+^CD4^+^ T cells was reduced in the cells pretreated with ATRA in both the 24 and 48 h pretreated groups.

To assess the effect of ATRA on Th17 T cells, naïve CD4^+^ T cells were stimulated in Th17 polarization conditions for 5 days to achieve strong Th17 polarization. Subsequently, cells were treated with either 1 × 10^−9^ m ATRA or no ATRA for 48 h (Figure S1c, Supporting Information). ATRA treatment significantly increased the fraction of FoxP3^+^ T cells and reduced the IL‐17A^+^ T cells (Figure S1d,e, Supporting Information). CD4^+^ T cell proliferation was comparable between the two groups (Figure S1f, Supporting Information).

To assess if similar ATRA concentrations could be effective in human T cells, a human Th17 polarization assay was conducted (Figure S1g,h, Supporting Information). Naïve human CD4^+^ T cells were isolated from peripheral healthy human donor blood, consistently obtaining greater than 90% CD4^+^CD45RO^−^CD62L^+^ post‐enrichment. Subsequently, these cells were stimulated with plate bound anti‐human CD3 and anti‐human CD28 antibodies along with IL‐6, TGF‐*β*1, IL‐1*β*, IL‐23, and IL‐21. ATRA was added to the human T cell expansion medium at concentrations ranging from 10 × 10^−12^ to 1 × 10^−9^ m. To represent the differential effect of ATRA on human T cells, the FoxP3^+^:IL‐17A^+^ T cell ratio was compared. At 1 × 10^−9^ m ATRA, the ratio of FoxP3^+^:IL‐17A^+^ cells in one donor was 0.99:1 ± 0.16, compared to 0.32:1 ± 0.05 and 0.36:1 ± 0.03 in no ATRA and 10 × 10^−12^ m ATRA, respectively (Figure S1i, Supporting Information).

The effect of ATRA on enhancing T_reg_ stability was assessed following a previously established T_reg_ destabilization assay.^[^ ^22^ ^]^ SKG splenocytes and lymphocytes were sorted by flow cytometry (TCR‐*β* ^+^CD4^+^eGFP^+^) to obtaining SKG FoxP3^eGFP+^ T_reg_ post‐sorting (Figure S1j, Supporting Information). FoxP3^eGFP^ T_reg_ were stimulated for 72 h with plate bound *α*CD3 and *α*CD28 with IL‐6 and 1 × 10^−9^ m ATRA added to the cell culture medium (Figure 1i). The addition of ATRA enhanced T_reg_ stability, with 70.8 ± 4.7% of cells retaining FoxP3^eGFP^ expression, significantly greater than 56.5 ± 2.2% cells retaining FoxP3^eGFP^ expression in the absence of ATRA (Figure 1j and Figure S1k, Supporting Information). The loss of FoxP3 expression correlated with T_reg_ transitioning to a Th17‐like ex‐T_reg_ phenotype, with 23.0 ± 1.7% and 13.3 ± 0.3% of cells without ATRA expressing ROR*γ*t and IL‐17A, respectively, compared to 11.6 ± 1.8% and 5.7 ± 1.1% of cells cultured with 1 × 10^−9^ m ATRA (Figure S1l, Supporting Information and Figure 1k,l). Treatment with ATRA resulted in comparable transcription factor and cytokine expression to T_reg_ not exposed to IL‐6 (Figure S1m–o, Supporting Information).

To measure the immunoregulatory effect of ATRA on other RA‐associated immune cells, differentiation experiments were conducted with SKG monocyte‐derived dendritic cells (DCs) and macrophages. Granulocyte‐macrophage colony‐stimulating factor (GM‐CSF) or macrophage colony‐stimulating factor (M‐CSF) was used to differentiate SKG bone marrow cells (BMCs) into DCs or macrophages, respectively (Figure S2a, Supporting Information). After 1 week of differentiation, DCs were stimulated with lipopolysaccharide (LPS), and macrophages were polarized using LPS and IFN*γ*. ATRA significantly reduced CD80 and CD86 expression by DCs compared to untreated stimulated DCs, at a level that was comparable to unstimulated DCs (Figure S2b–d, Supporting Information). In DCs, MHCII expression was comparable in all conditions (Figure S2e, Supporting Information). ATRA significantly reduced TNF expression by macrophages compared to untreated stimulated macrophages at a level that was comparable to unpolarized macrophages (M0) (Figure S2f,g, Supporting Information). MHCII expression between ATRA‐treated and untreated polarized macrophages was comparable and higher than the unstimulated macrophages not treated with LPS (Figure S2h, Supporting Information).

### 2.2 ATRA Promotes a Unique Chromatin Landscape in T Cells Differentiated in Th17 Polarizing Conditions

**Figure 2:**
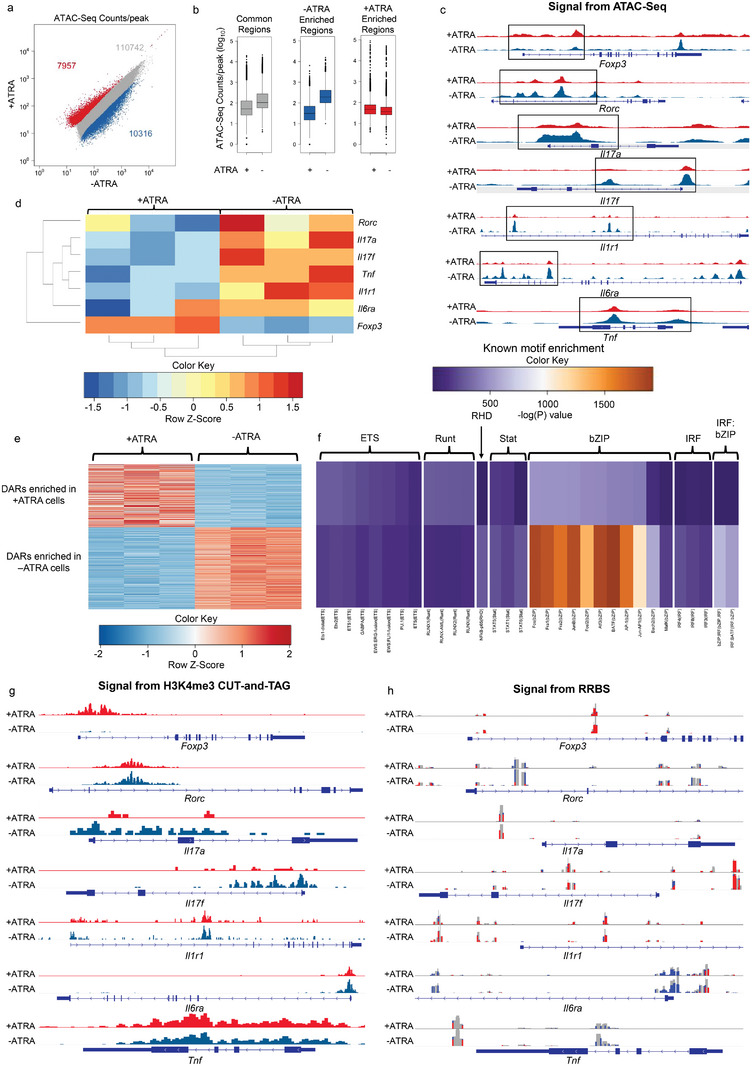
ATRA differentially modulates chromatin accessibility at Th17 and T_reg_ associated loci in Th17 polarizing conditions. a) Scatterplots of ATAC‐Seq counts per peak comparing cells exposed to Th17 polarizing conditions treated with 1 × 10^−9^ m ATRA (+ATRA, *n* = 3) or without ATRA (−ATRA, *n* = 3). Experimental timeline was the same as that depicted in Figure 1a. Red corresponds to DARs enriched in the +ATRA condition, blue corresponds to DARs enriched in the −ATRA condition, and gray corresponds to common regions that are accessible in both conditions, but not significantly different. b) Boxplots of ATAC‐Seq counts per peak from +ATRA and −ATRA conditions at common (gray) or DARs enriched in either the +ATRA (red) or the −ATRA group (blue) from the comparison in (a). Boxes indicate interquartile range with whiskers ± 1.5 times this range and outlier points. c) Normalized ATAC‐Seq coverage at the *Foxp3*, *Rorc*, *Il17a*, *Il17f, Il1r1, Il6ra*, and *Tnf* loci in average representations of the +ATRA and −ATRA conditions. d) Heatmap of select Th17 and T_reg_ associated genes visualized in (c) for −ATRA and +ATRA conditions with dendrograms showing relatedness of samples (columns) and individual genes (rows). e) Heatmap of all DARs quantified by *z*‐score. DARs enriched in the +ATRA condition correspond to the red dots in (a), while DARs enriched in the −ATRA condition correspond to the blue dots in (a). f) Motif enrichment analysis for the grouped DARs in (e) quantified by enrichment of motif across clusters shown in (e) with enrichment quantified as the negative of the log(*P*) value. g) H3 Histone lysine 4 trimethylation (H3K4me3) coverage analyzed using CUT‐and‐TAG and h) CpG methylation patterns determined via RRBS at the same loci as in (c) in representative samples from the +ATRA and −ATRA conditions. All representative RRBS regions of interest are zoomed‐in to improve clarity in promoter regions except *Tnf*. Scales in (c): *Foxp3* [0‐150], *Rorc* [0‐380], *Il17a* [0‐130], *Il17f* [0‐260], *Il1r1* [0‐700], *Il6ra* [0‐920], *Tnf* [0‐900]; boxes in (c) highlight regions of apparent signal differences between +ATRA and −ATRA cells at respective gene loci; data in (d) represent the integrated ATAC signal in normalized reads across each known gene promoter region ranked as *z*‐scores using data across each row; data in (e) represent DAR *z*‐scores calculated using the average of all samples, i.e., across each row; data in (f) represent motifs with an enrichment log(*P*) value less than −35 found in 10% or more regions with coverage showing a fold increase of at least 1.5 over background coverage in at least one cluster; scales in (g): *Foxp3* [0‐31], *Rorc* [0‐123], *Il17a* [0‐2.5], *Il17f* [0‐5], *Il1r1* [0‐5], *Il6ra* [0‐72], *Tnf* [0‐45]; scales in h: *Foxp3* [0‐70], *Rorc* [0‐26], *Il17a* [0‐26], *Il17f* [0‐12], *Il1r1* [0‐26], *Il6ra* [0‐76], *Tnf* [0‐79].

The enhancement of T_reg_ generation in Th17 polarizing conditions was correlated with changes in chromatin accessibility. Naïve SKG CD4^+^ T cells were used in a Th17 polarization assay, described in Figure 1a, and the chromatin accessibility profiles of cells treated with 1 × 10^−9^ m ATRA (+ATRA) or cells in Th17 polarizing conditions alone (−ATRA) were compared by assay for transposable‐accessible chromatin with high‐throughput sequencing (ATAC‐Seq). Approximately 18 000 differentially accessible regions (DARs) were found, with ≈8000 regions more accessible in the +ATRA condition, and ≈10 000 regions more accessible in the −ATRA condition (**Figure** **2**a,b). The accessible chromatin pattern between the two conditions was notably distinct at the *Foxp3*, *Rorc*, *Il17a*, *Il6rRa*, and *Il1r1* loci, which are relevant for T_reg_ and Th17 phenotypes (Figure 2c). The integrated chromatin accessibility of the *Foxp3* promoter region was enhanced in the +ATRA condition while that of the *Rorc*, *Il17a*, *Il6rRa*, and *Il1r1* promoter regions was reduced or nearly absent in the +ATRA relative to the −ATRA condition (Figure 2d).

Chromatin interactions maintain contact between genes and distal regulatory elements, such as enhancers and promoters. To confirm potential molecular mechanisms of action of ATRA, the above‐mentioned chromatin landscape data were interrogated for transcription factor (TF) binding motifs that were differentially accessible between the groups (Figure 2e,f). A differential motif enrichment analysis showed that DARs enhanced in the +ATRA condition were enriched in Runt family motifs and select ETS family TFs such as Ets1 and Etv2. Members of the ETS family have been associated with promoting the development of T_reg_ as well as enhancing their stability and function.^[^ ^23^, ^24^ ^]^ Members of the Runt family of transcription factors have been shown to have pleiotropic effects in T_reg_ differentiation. Differential association of Runx1 with FoxP3 or Ror*γ*t can inhibit or promote Th17 differentiation, respectively, and binding of a complex of Runx1 and core‐binding factor subunit beta (CBF*β*) promotes FoxP3 expression in T_reg_.^[^ ^25^ ^]^ The DARs enhanced in the −ATRA condition were enriched in binding motifs for select bZIP family transcription factors including JunB and BATF which are associated with enhanced Th17 differentiation.^[^ ^26^, ^27^ ^]^

To assess potential factors that affect chromatin and gene accessibility, modifications to histone and CpG methylation patterns were analyzed. The Cleavage Under Targets and Tagmentation (CUT‐and‐TAG) assay was conducted to assess the degree of trimethylation at histone H3 lysine 4 (H3K4me3), an epigenetic modification often found at promoter sites known for enhancing transcription, in naïve SKG CD4^+^ T cells differentiated in +ATRA and −ATRA conditions, as above.^[^ ^28^, ^29^ ^]^ Significant differences in H3K4me3 modifications were found at the *Foxp3* locus with an ≈12‐fold change in signal in +ATRA cells compared to −ATRA cells, as well as other sites implicated in T_reg_ function and stability, including *Hic1*, *Cd38*, and *Bcl6* ^[^ ^30^, ^31^, ^32^, ^33^ ^]^ (Figure 2g and Figure S3a, Supporting Information). However, there were no substantial differences in H3K4me3 methylation patterns at Th17‐associated gene loci including *Rorc* and *Tnf*. As hypomethylation of the CNS2 region of *Foxp3* is known to be associated with natural T_reg_ (nT_reg_) stability, the CpG methylation patterns were analyzed for differential methylation of DNA using reduced representation bisulfite sequencing (RRBS). No significant changes to CpG methylation were seen between +ATRA and −ATRA treated cells in the promoter regions of representative T_reg_ and Th17‐associated loci (Figure 2h). Further, only six differentially methylated regions were observed between samples, and the regions have not been associated with T_reg_ function or stability (Figure S3b, Supporting Information).

### 2.3 PLGA MPs Sustain Bioactive ATRA Release

**Figure 3:**
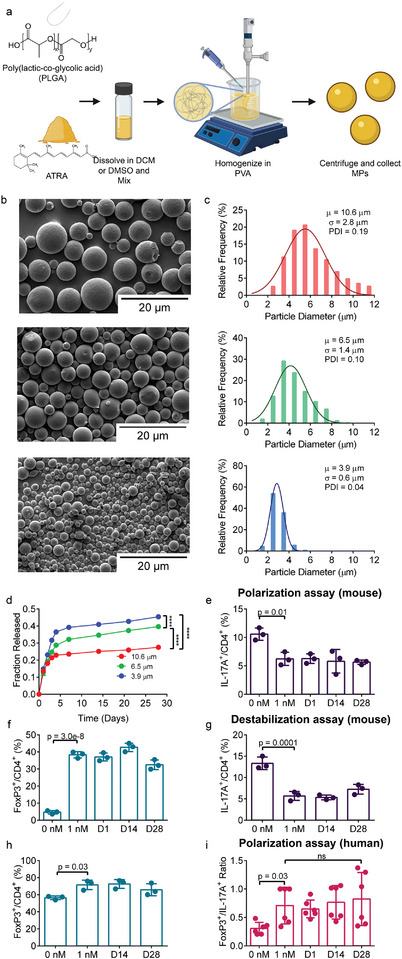
Sustained release of bioactive ATRA from PLGA MP. a) Experimental schematic for formulating PLGA‐ATRA MP. b) Scanning electron micrographs (SEMs) of PLGA‐ATRA MP c) spanning varying size distributions. Measurements are based on image analysis outlined in Figure S4a in the Supporting Information. d) Release of ATRA from 10 mg of 10.6, 6.5, or 3.9 µm PLGA‐ATRA MP over 28 days. e,f) Bioactivity of post‐release ATRA measured collected at different time intervals on CD4^+^ mT cell differentiation as measured by effect e) IL‐17A expression and f) FoxP3 expression. g,h) Bioactivity of ATRA released from PLGA‐ATRA MP formulations in mT_reg_ destabilization as measured by g) IL‐17A expression and h) FoxP3 expression. i) PLGA‐ATRA MP release supernatant tested in healthy human (h)T cell differentiation assays described in Figure S1 in the Supporting Information. Averages in (c) are based on averaging the analysis of three batches per size; data in (c) are distributions and diameter averages from a single batch for each size, compiled from analysis of three different image sections per size; size distribution analysis represented in (c) was performed for two additional batches for each size and similar results were obtained (Figure S4b, Supporting Information); data in (d)–(i) are the means ± SD of technical replicates from representative experiments; data in (d) are representative of three technical replicates performed using a single batch of particles for each size; (e)–(h) were performed with two experimental replicates; data in (i) are representative of two donors using three technical replicates per donor. Statistical analysis in (d) was performed by comparing the area under the curve for each replicate using a one‐way ANOVA with post hoc Tukey test, ****p < 0.0001; analyses in (e)–(i) were performed using ANOVA with Tukey's multiple comparison test. Schematic in (a) was composed using BioRender and ChemDraw.

The aforementioned results suggested that ATRA is well‐suited as a potential IA inducer of persistent immunoregulatory changes that could be transferred to other affected joints via systemic recirculation of T cells. To this end, IA injectable microparticles were developed from PLGA using a single emulsion method to generate PLGA‐ATRA MP (**Figure** **3**a). Scanning electron microscopy of lyophilized PLGA‐ATRA MP was used to characterize the surface morphology. In general, the surface of pristine PLGA‐ATRA MP was uniformly textured (Figure 3b). By controlling the homogenization rate, particles with differing size ranges were generated. The volume‐averaged particle size across three batches was quantified (Figure S4a,b, Supporting Information). The polydispersity index within each batch was less than 0.30 in all conditions (Figure 3c). The loading efficiency of ATRA into the MPs was 62.4 ± 3.2% which resulted in PLGA‐ATRA MP with a composition of ≈1.2 wt% ATRA.

To quantify ATRA release in vitro, 10 mg PLGA‐ATRA MP were suspended in 1 mL of 0.1% bovine serum albumin (BSA) in phosphate‐buffered saline (PBS) and incubated at 37 °C, collecting release supernatant over 28 days at predetermined timepoints. Approximately 13% of ATRA released within the first 24 h (Figure 3d). From 24 to 96 h, 10 ± 0.6%, 15 ± 0.2%, and 22 ± 1.3% of the original ATRA content was released from the 10.6, 6.5, and 3.9 µm average diameter PLGA‐ATRA MP, respectively. Subsequently, ATRA release was sustained from all PLGA‐ATRA MP for the next 24 days at a rate of ≈0.4% of the initial loaded ATRA per day, corresponding to a release of ≈0.52 ng ATRA per mg of particles per day. To characterize changes in particle morphology during the course of in vitro degradation, PLGA‐ATRA MP were periodically collected, washed, and imaged. Dynamic morphological changes in the particle structure were observed which included an initial phase of swelling, erosion, and final‐stage structural decomposition (Figure S4c,d, Supporting Information). At day 21, 10.6 µm particles had eroded but retained morphology while the 6.5 µm particles had undergone evident erosion and structural changes and most 3.9 µm particles had decomposed.

The bioactivity of released ATRA was assayed following the experimental schematic outlined in Figure 1a with supernatant collected at day 1, 14, and 28 from 6.5 µm particles, diluted to the expected joint concentration based on delivery of 2 µg PLGA‐ATRA MP injected in ≈20 µL of synovial fluid. In a mouse Th17 polarization assay, released ATRA collected at all three timepoints and freshly prepared 1 × 10^−9^ m ATRA comparably increased FoxP3 expression and reduced IL‐17A expression (Figure 3e,f). Released ATRA also maintained T_reg_ stability comparable to 1 × 10^−9^ m ATRA, improving FoxP3 expression while suppressing IL‐17A expression (Figure 3g,h). In a human Th17 polarization assay, released ATRA also retained bioactivity comparable to 1 × 10^−9^ m ATRA (Figure 3i).

To estimate in vivo concentrations of ATRA in the synovial fluid, spleen, and peripheral blood after IA injection, a two‐compartment pharmacokinetic model was developed based on the in vitro release profile (Figure S5a, Supporting Information). The biodistribution of ATRA was approximated using first‐order rate equations and previously reported experimentally measured kinetic parameters for ATRA half‐life in the serum and synovium permeability data (Figure S5b, Supporting Information).^[^ ^34^, ^35^ ^]^ The model showed that after an initial spike, concentrations in the joint would be maintained at greater than 6 × 10^−9^ m for at least 28 days with 6.5 µm PLGA‐ATRA MP, while the concentration in the peripheral blood are below physiologically relevant values (<20 × 10^−12^ m) (Figure S5c–e, Supporting Information). Based on the model and degradation profile, the 6.5 µm PLGA‐ATRA MP was selected for further evaluation.

### 2.4 PLGA‐ATRA MP Suppress Joint Inflammation in SKG Arthritis

**Figure 4:**
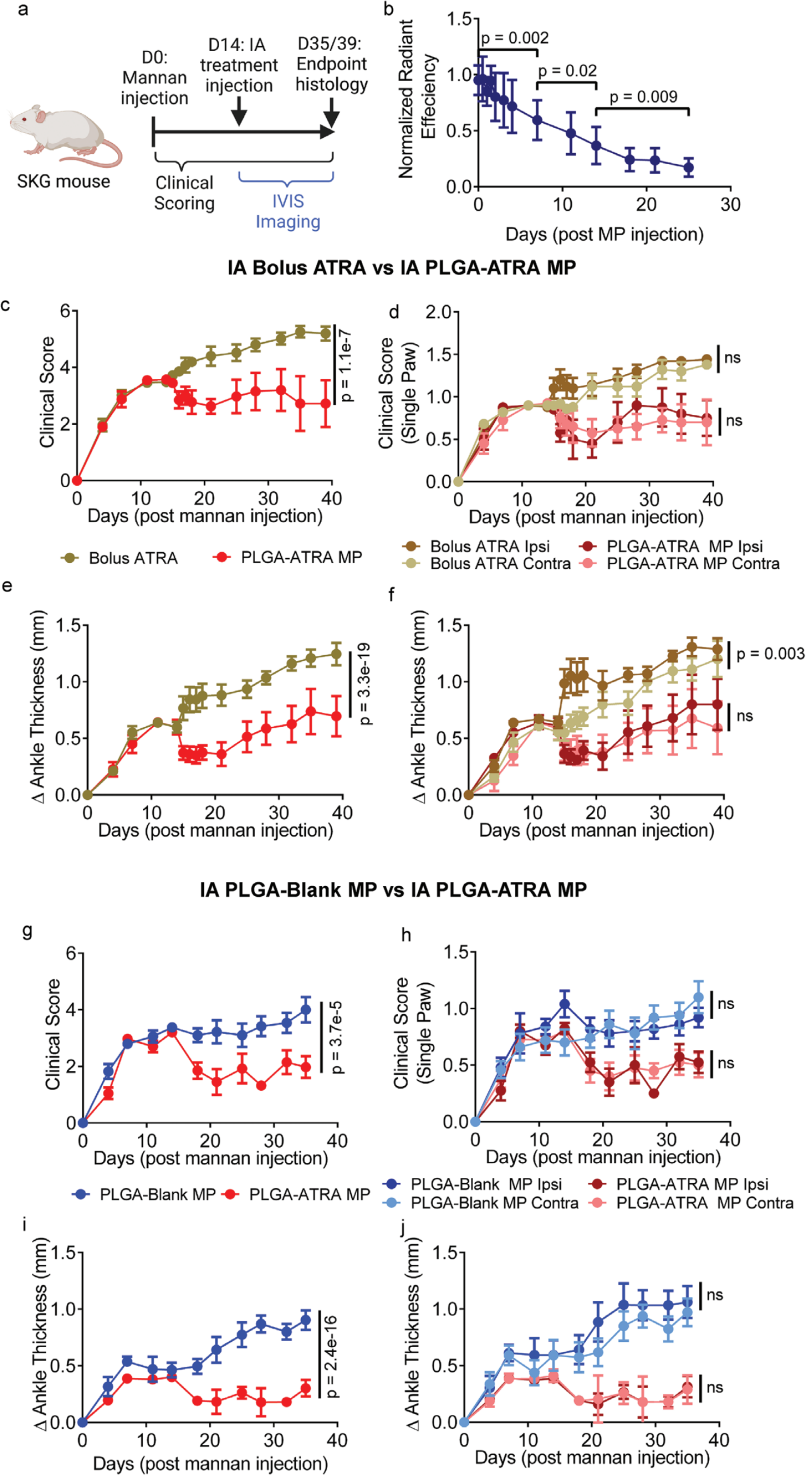
PLGA‐ATRA MP modulates autoimmune arthritis in SKG mice. a) Experimental schematic for assessing the efficacy of PLGA‐ATRA MP in treating established arthritis. b) Normalized fluorescence signal from IA injected Cy5‐tagged PLGA‐Blank MP, quantified using an IVIS. c–f) Clinical arthritis scores in mice treated with either IA PLGA‐ATRA MP or dose‐matched bolus IA ATRA presented as c) aggregated clinical scores, d) disaggregated ipsilateral (Ipsi) and contralateral (Contra) hind paws, e) aggregated ankle thickness measurements of hind paws, and f) disaggregated ankle thickness measurements of ipsilateral and contralateral hind paws. g–j) Clinical arthritis scores in mice treated with either IA PLGA‐ATRA MP or PLGA‐Blank MP presented as g) aggregated clinical scores, h) disaggregated ipsilateral and sham PBS‐injected contralateral hind paws, i) aggregated ankle thickness of hind paws, and j) disaggregated ankle thickness measurements of ipsilateral and contralateral hind paws. Data in (b) are means ± SD of normalized radiant efficiency of Cy5‐tagged PLGA‐Blank MP in *n* = 9 mice; data in (c)–(j) are the means ± SEM; data in (c, e) share a legend, data in (d, f) share a legend, data in (g, i) share a legend, data in (h, j) share a legend; (c–f) (*n* = 9 mice for PLGA‐ATRA MP, *n* = 9 mice for bolus IA ATRA) represent data from two independent experiments and (g–j) (*n* = 9 mice for PLGA‐ATRA MP, *n* = 11 mice for PLGA‐Blank MP) represent data from four independent experiments. Statistical analyses in (b) were performed using a repeated measures one‐way ANOVA with post hoc Sidak's test for multiple comparisons; statistical analyses in (c–j) were performed using a linear mixed‐effects model; schematic in (a) was composed using BioRender.

SKG mice develop spontaneous RA‐mimicking polyarthritis, and the onset can be accelerated by injections of fungal components.^[^ ^36^, ^37^ ^]^ To assess the feasibility of modulating established SKG arthritis, PLGA‐ATRA MP were IA‐injected in the ankle of SKG mice with mid‐stage arthritis. Arthritis onset was synchronized using i.p. mannan injection and the treatment was administered after 14 days (**Figure** **4**a). To measure particle persistence in the joint, cyanine‐5 (Cy5) conjugated PLGA was incorporated to generate fluorescently labeled Cy5 PLGA‐ATRA MP and PLGA MP without ATRA (PLGA‐Blank MP) and quantified the Cy5 signal in live animals using an in vivo imaging system (IVIS).

Post‐IA injection, the Cy5 signal remained localized in the joint and steadily decreased in intensity over the course of the study (Figure 4b and Figure S6a, Supporting Information). As the Cy5 signal from deeper tissues could be attenuated, harvested tissues from additional cohorts of mice were imaged at 1 and 5 days post‐IA injection. No signal above background was detected in the draining lymph nodes, kidneys, liver, or spleen at either timepoint (Figure S6b, Supporting Information). To quantify uptake of particles by immune cells, flow cytometry analysis of the homogenized ankles, draining lymph nodes, and spleen was conducted (Figure S6c, Supporting Information). Cy5 was detected primarily in CD45^+^CD11b^+^CD11c^−^ and CD45^+^CD11b^+^CD11c^+^ cells isolated from the injected ankle, with minimal uptake in CD4^+^ cells, and no detection outside the injected ankle (Figure S6d, Supporting Information).

To assess the role of the sustained release of IA ATRA in suppressing SKG arthritis, IA PLGA‐ATRA MP were compared to a dose‐matched IA injection of bolus ATRA. Mice received 2 µg PLGA‐ATRA MP suspended in 20 µL of sterile PBS in a single hind ankle joint (ipsilateral ankle) via IA injection (Figure S7a, Supporting Information). In a separate group of mice, dose‐matched bolus ATRA in corn oil was injected in the ipsilateral ankle. In both groups, the opposite ankle joint (contralateral ankle) received a sham injection of PLGA MP without ATRA (PLGA‐Blank MP). Arthritis progression was compared between the treatment groups using bi‐weekly clinical scoring, following a previously established method.^[^ ^38^ ^]^ The pretreatment clinical scores of mice were comparable between the groups, which increased rapidly from an initial score of 0 to an average score of 3.5 on day 14 prior to treatment with mild swelling in both wrist and ankle joints, with the majority of the digits swollen (Figure 4c–f). Post‐treatment, arthritis was suppressed in IA PLGA‐ATRA MP treated mice with strong reductions in inflammation in all joints, but no clinical benefit was seen in mice treated with a dose‐matched IA bolus ATRA.

To confirm that the antiarthritic effect was due to the ATRA from the PLGA‐ATRA MP, IA PLGA‐ATRA MP were compared with PLGA‐Blank MP. Both groups had an average score of 3.5 on day 14 prior to treatment, as above (Figure 4g). Clinical scores decreased in mice following treatment with a single IA injection of 2 µg PLGA‐ATRA MP, but not PLGA‐Blank MP, 4 days (D18: 1.9 ± 0.4) and 1 week (D21: 1.4 ± 0.5) post‐treatment and remained reduced until the study endpoint (D35: 1.9 ± 0.5). The scores were significantly lower than those in PLGA‐Blank MP‐treated mice measured at the same timepoints (D18: 3.1 ± 0.5, D21: 3.2 ± 0.9, D35: 4.0 ± 1.0). In addition to the 2 µg PLGA‐ATRA MP‐ and PLGA‐Blank MP‐treated mice, a subset of mice received a higher dose, either 20 or 200 µg of PLGA‐ATRA MP to assess if there was a dose‐dependent effect in vivo. Clinical scores of all groups treated with PLGA‐ATRA MP were comparable (Figure S7b, Supporting Information). Arthritis scores in all mice that received PLGA‐ATRA MP decreased and stabilized following treatment for the remainder of the study. In contrast, the clinical scores in PLGA‐Blank MP‐treated mice progressively increased until the study endpoint at day 35. The improvement in clinical score and ankle thickness measurements in PLGA‐ATRA MP‐treated mice was quantified in both the ipsilateral and contralateral joints (Figure 4h–j and Figure S7c,d, Supporting Information). Ankle thickness of the hind paws in 2 µg PLGA‐ATRA MP‐treated mice remained stable or decreased following treatment. In contrast, clinical scores increased comparably in both the ipsilateral and contralateral ankles in IA bolus ATRA and PLGA‐Blank MP‐treated mice. To confirm the clinical observations of PLGA‐ATRA MP‐mediated, inflammatory markers in the ipsilateral and contralateral joint were quantified. Quantitative polymerase chain reaction (qPCR) confirmed that the inflammatory markers *Il6*, *Il1b*, *Tnf*, *Mmp3*, *Mmp13* were reduced in both the ipsilateral and contralateral joints of mice that received PLGA‐ATRA MP but not PLGA‐Blank MP, while *Tgfb1* was comparable between groups (Figure S8a–g, Supporting Information).

### 2.5 PLGA‐ATRA MP Decreases Synovial Infiltrates, Cartilage Damage, and Bone Erosions

**Figure 5:**
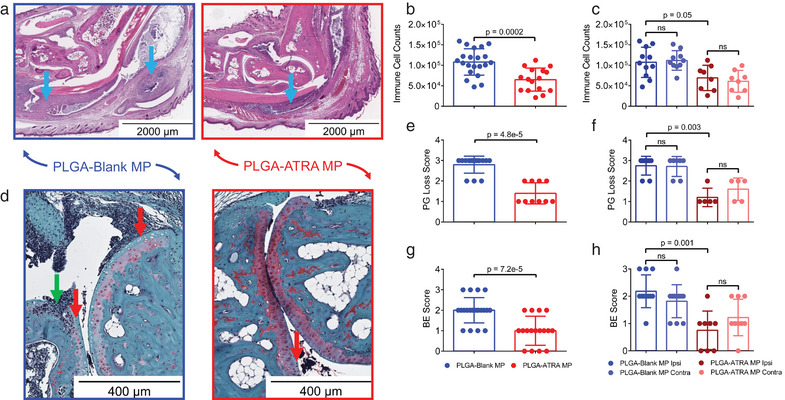
PLGA‐ATRA MP reduce immune cell infiltration, cartilage damage, and bone erosions in treated mice. a) Representative H&E‐stained histology sections of ankles from arthritic SKG mice, treated as depicted in Figure 4a with either PLGA‐ATRA MP or PLGA‐Blank MP. Immune cells (blue arrows) were quantified and are reported as b) aggregated infiltration in ankle joints and c) disaggregated infiltration in ipsilateral (Ipsi) and contralateral (Contra) ankles. d) Representative safranin‐O‐stained histological sections showing bone erosion (green arrow) and proteoglycan loss (red arrows) at the talus/tibia junction. e,f) Quantification of proteoglycan (PG) loss reported as e) aggregated scores from both ankles and f) disaggregated scores in ipsilateral and contralateral ankles. g,h) Quantification of bone erosion reported as g) aggregated scores from both ankles and h) disaggregated scores in ipsilateral and contralateral ankles. Data in (b), (c), and (e–h) represent the mean ± SD. Data for bone erosion scoring and immune cell counts are from *n* = 21 sections from 11 mice for the PLGA‐Blank MP group and *n* = 16 sections from 9 mice for the PLGA‐ATRA MP group. Data for proteoglycan loss are from *n* = 14 sections from seven mice for the PLGA‐Blank MP group and *n* = 10 sections from five mice for the PLGA‐ATRA MP group. Data for bone erosion are from *n* = 22 sections from 11 mice for the PLGA‐Blank MP group and *n* = 16 sections from nine mice for the PLGA‐ATRA MP group. Statistical analysis in (b) was performed using unpaired Student's *t*‐test; analysis in (c) was performed using a one‐way ANOVA with post hoc Tukey test for multiple comparisons; statistical analysis in (e) and (g) was performed using a Mann–Whitney test; statistical analyses in (f) and (h) were performed using Kruskal–Wallis test with a post hoc Dunn's multiple comparison test.

To assess the structure of arthritic SKG joints, ipsilateral and contralateral ankles from PLGA‐Blank MP and PLGA‐ATRA MP‐treated mice (subject to the analysis as described in Figure 4) were processed for histology after sacrifice on day 35. Hematoxylin and eosin (H&E)‐stained sections of arthritic ankle sections showed reduced inflammation in the joints of PLGA‐ATRA MP‐treated mice compared to joints from PLGA‐Blank MP‐treated mice (**Figure** **5**a). Inspired by the guidelines recently published for Standardized Microscopic Arthritis Scoring of Histological sections (SMASH), a computer‐aided algorithm in the QuPath software was generated using default settings for tissue thresholding and cell detection/classification, to facilitate quantification of cell infiltrates.^[^ ^39^ ^]^ Ankles from mice treated with 2 µg PLGA‐ATRA MP had significantly reduced cellularity compared to mice that received PLGA‐Blank MP and bolus ATRA (Figure 5b and Figure S9a–c, Supporting Information). The synovial inflammation and infiltration were comparable between the contralateral and ipsilateral hind joints of the same treatment groups (Figure 5c and Figure S9d, Supporting Information). Cartilage proteoglycan (PG) loss and bone erosion (BE) scoring was performed on SMASH‐recommended safranin‐O‐stained ankle joint sections from PLGA‐Blank MP‐, bolus ATRA‐, and 2 µg PLGA‐ATRA MP‐treated mice. The PG loss scores for PLGA‐Blank MP‐treated ankles (2.8 ± 0.4) and bolus ATRA‐treated ankles (2.5 ± 0.5) were both higher than in 2 µg PLGA‐ATRA MP‐treated ankles (1.4 ± 0.5) while 20 and 200 µg PLGA‐ATRA MP treated mice had comparable PG loss scores to the 2 µg PLGA‐ATRA MP mice (Figure 5d,e and Figure S9e,f, Supporting Information). PG loss scores were comparable between the ipsilateral and contralateral ankles of the PLGA‐Blank MP and 2 µg PLGA‐ATRA MP treatment groups (Figure 5f). 2 µg PLGA‐ATRA MP‐treated ankles had a BE score of 1.0 ± 0.7, while PLGA‐Blank MP‐treated ankles had a BE score of 2.0 ± 0.6 and bolus ATRA‐treated ankles had a BE score of 2.6 ± 0.5 (Figure 5g and Figure S9g, Supporting Information). BE scores were comparable between ipsilateral and contralateral ankles of the PLGA‐Blank MP and 2 µg PLGA‐ATRA MP treatment groups (Figure 5h).

### 2.6 IA PLGA‐ATRA MP Suppress Th17 and Enhance T_reg_ at Local Sites of Inflammation

**Figure 6:**
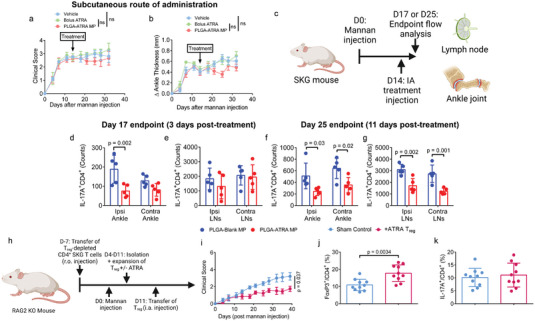
IA PLGA‐ATRA MP modulate T cells in joints and draining lymph nodes to promote systemic disease modulation. a,b) Quantification of arthritis progression using a) clinical scores and b) ankle thickness measurements in mice treated subcutaneously (SubQ) with PLGA‐ATRA MP (*n* = 6), single dose‐matched bolus ATRA (*n* = 5) or vehicle (*n* = 6). c) Experimental schematic for immunophenotyping T cells in SKG mice after IA treatment. d,e) IL‐17A^+^CD4^+^ T cells in the ipsilateral (Ipsi) and contralateral (Contra) d) ankles and e) draining lymph nodes 3 days post‐ IA PLGA‐ATRA MP or IA PLGA‐Blank MP. f,g) IL‐17A^+^CD4^+^ cells in the f) ipsilateral (Ipsi) and contralateral (Contra) ankles and g) draining lymph nodes 11 days post‐ IA PLGA‐ATRA MP or IA PLGA‐Blank MP. h) Experimental schematic for transfer of arthritogenic and regulatory T cells in RAG2‐KO mice. i) Quantification of arthritis progression using clinical scores. Endpoint ankle‐isolated j) FoxP3^+^CD4^+^ T cells and k) IL‐17A^+^CD4^+^ T cells. Data in (a, b) (Vehicle *n* = 6, dose‐matched bolus ATRA *n* = 5, PLGA‐ATRA MP *n* = 6) represent mean ± SEM; data in (d–g) (*n* = 5 per group) represent mean ± SD; data in (i) represent mean ± SEM (*n* = 5 per group); data in (j, k) represent the mean ± SD. Statistical analyses in (a, b, i) were performed using a linear mixed effect model analysis. Statistical analyses in (d–g) were performed using a one‐way ANOVA with post hoc Sidak's test. Statistical analyses in (j, k) were performed using an unpaired Student's two tailed *t*‐test. Schematics in (a) and (i) were composed using BioRender.

We sought to assess whether the IA route of administration uniquely contributed to the systemic antiarthritic effect of PLGA‐ATRA MP. To this end, arthritic SKG mice were treated with either 2 µg PLGA‐ATRA MP, dose‐matched bolus ATRA, or 2 µg PLGA‐Blank MP administered subcutaneously into the midscapular scruff region. Unlike with IA PLGA‐ATRA MP, no significant improvement in clinical scores or ankle thickness measurements was observed in any of the treatment groups (**Figure** **6**a,b).

To characterize local and systemic immune modulation mediated by IA PLGA‐ATRA MP, CD4^+^ T cell subsets in the ankles, draining lymph nodes, and spleen were characterized 3 days and 11 days post‐IA‐PLGA‐ATRA MP treatment (Figure 6c and Figure S10a, Supporting Information). 3 days post‐treatment, the number of infiltrating Th17 cells modestly decreased at 3 days in the ipsilateral and contralateral ankles of mice that received 2 µg PLGA‐ATRA MP compared to mice that received PLGA‐Blank MP (Figure 6d) but not in the draining lymph nodes or spleens (Figure 6e and Figure S10b, Supporting Information). 11 days post‐treatment, the number of Th17 in both the ipsilateral and contralateral ankles was lower in mice that received PLGA‐Blank MP compared to mice that received PLGA‐ATRA MP (Figure 6f). The same trend was observed in the pooled draining lymph nodes (inguinal and popliteal), with higher Th17 cell counts in both the ipsilateral ankle‐draining lymph nodes as well as the contralateral ankle‐draining lymph nodes of PLGA‐Blank MP‐treated mice relative to PLGA‐ATRA MP‐treated mice (Figure 6g). No difference was observed in the spleens of mice between treatment groups (Figure S10c, Supporting Information).

We sought to validate the role of ATRA‐enhanced T_reg_ in mediating arthritis protection. SKG CD4^+^ T cells can transfer arthritis to RAG2‐KO mice.^[^ ^40^ ^]^ Following a previously established assay, T_reg_‐depleted SKG T cells (CD4^+^CD25^−^) were isolated and transferred to RAG2‐KO mice via retro‐orbital injection.^[^ ^22^ ^]^ Subsequently, these mice were injected with mannan to induce arthritis. Arthritic mice were then treated via monoarticular injection with either T_reg_ differentiated ex vivo from naive CD4^+^ T cells in media containing IL‐2, TGF‐*β*, and 1 × 10^−9^ m ATRA (+ATRA T_reg_), or a sham PBS control (Figure 6h). Mice that received +ATRA T_reg_ had significantly reduced clinical scores relative to mice receiving sham PBS (Figure 6i). The T_reg_ fraction was higher in the ankles of mice that received +ATRA T_reg_, while the IL‐17A^+^ fraction of T cells was comparable (Figure 6j,k). Moreover, ATRA enhanced CCR9 but not CCR6 expression in ex vivo differentiated T_reg_ (Figure S11a,b, Supporting Information).

**Figure 7:**
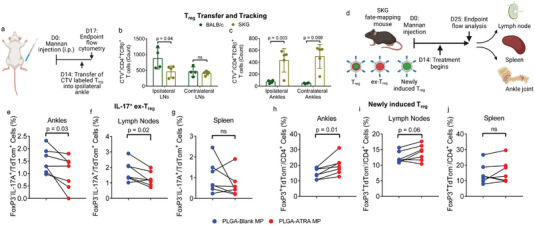
IA PLGA‐ATRA MP promote T_reg_ stability and differentiation in vivo. a) Experimental schematic for tracking IA injected T_reg_. b,c) Quantification of CellTracker Violet^+^ (CTV^+^) CD4^+^TCR‐*β* ^+^ T cells in the b) lymph nodes and c) ankles 3 days post‐IA injection. d) Experimental schematic for measuring T_reg_ stability and differentiation in SKG fate‐mapping mice. e–g) Percent of destabilized T_reg_ (FoxP3^eGFP−^IL‐17A^+^/TdTom^+^) in the e) ankles, f) draining lymph nodes, and g) spleens of littermate paired fate‐mapping SKG mice. h–j) Percent of newly induced T_reg_ (FoxP3^eGFP+^tdTom^−^) in the h) ankles, i) draining lymph nodes, and j) spleen 32 days after cessation of tamoxifen administration in paired littermates that are treated with either IA PLGA‐Blank MP or PLGA‐ATRA MP. Data in (b, c) (BALB/cJ *n* = 4, SKG *n* = 5) are represent mean ± SD of two representative experiments; data points in (e–j) represent individual mice, with paired littermates treated with either PLGA‐Blank MP (*n* = 7) or PLGA‐ATRA MP (*n* = 7) compared. Statistical analyses in (b, c) were performed using one‐way ANOVA with post hoc Tukey test. Statistical analyses in (e–j) were performed using a paired Student's two‐tailed *t*‐test. Schematics in (a) and (d) were composed using BioRender.

To assess whether T_reg_ recirculation between joints would phenocopy the systemic control of disease observed after monoarticular injection of PLGA‐ATRA MP. SKG FoxP3^eGFP^ T_reg_, isolated as described above (Figure 1i and Figure S11c, Supporting Information), were expanded for 7 days. Subsequently, the cells were labeled with CellTracker Violet (CTV) and transferred, via monoarticular ankle IA injection, into either nonarthritic BALB/cJ mice or arthritic SKG mice (**Figure** **7**a). 3 days post‐injection, mice were sacrificed and CTV^+^ T_reg_ were quantified in the spleen, draining lymph nodes, contralateral lymph nodes, and contralateral and distal paws (Figure S11d, Supporting Information). CTV^+^ T_reg_ were detected in all analyzed tissues with a preferential accumulation in both the ipsilateral and contralateral arthritic joints of SKG mice, whereas the majority of CTV^+^ T_reg_ were found in the pooled inguinal and popliteal draining lymph nodes or the spleen of BALB/cJ mice (Figure 7b,c and Figure S11e, Supporting Information). The number of CTV^+^ T_reg_ in the contralateral lymph nodes was comparable between arthritic SKG mice and BALB/cJ mice. IA administration of T_reg_ also reduced overall arthritis severity as measured by clinical scores in arthritic SKG mice and decreased the thickness of the contralateral ankles (Figure S11f,g, Supporting Information). Strikingly, the number of CTV^+^ T_reg_ in the injected joint of the arthritic mice was comparable to that in the pooled distal joints, supporting that recirculation of arthritis relevant T_reg_ from the joint can mediate arthritis protection.

As T_reg_ instability is known to exacerbate disease in SKG mice, we sought to assess whether PLGA‐ATRA MP promote the stability of T_reg_ and the differentiation of new T_reg_ in vivo. A previously established T_reg_ fate‐mapping SKG mouse model was used in which cells actively expressing FoxP3 also express enhanced green fluorescent protein (eGFP), and cells that expressed FoxP3 during tamoxifen administration also express tdTomato (tdTom), allowing for the identification of stable T_reg_ (CD4^+^tdTom^+^FoxP3^eGFP+^IL‐17A^−^) and ex‐T_reg_ (CD4^+^tdTom^+^FoxP3^eGFP−^IL‐17A^+^) (Figure 7d)_._^[^ ^41^ ^]^ As tamoxifen administration ends 1 week prior to arthritis induction, the model also allows for the identification of newly induced T_reg_ (CD4^+^tdTom^−^FoxP3^eGFP+^). To assess the effect of PLGA‐ATRA MP on T_reg_ stability, CD4^+^tdTom^+^ T cells after arthritis induction were compared between littermates that received IA PLGA‐ATRA MP or PLGA‐Blank MP, analyzing the pooled draining lymph nodes (inguinal and popliteal), pooled ankles, and the spleen. The reduced arthritis severity in this model necessitated pooling of the joints and lymph nodes to obtain adequate number of cells for analysis. Littermates that received PLGA‐ATRA MP had a reduced fraction of ex‐T_reg_ in both joint draining lymph nodes and ankles as compared to littermates that received PLGA‐Blank MP, while the fraction of ex‐T_reg_ in the spleen was comparable between treatment groups (Figure 7e–g). To assess the effect of PLGA‐ATRA MP on in situ T_reg_ induction, the fraction of CD4^+^tdTom^−^FoxP3^eGFP+^ T cells in the draining lymph nodes, ankles, and spleen was analyzed. The fraction of induced T_reg_ was increased in the ankles of PLGA‐ATRA MP‐treated littermates (Figure 7h). The fraction of induced T_reg_ in the draining lymph nodes of mice that received PLGA‐ATRA MP was modestly increased in five of the seven pairs, but this trend was not statistically significant (Figure 7i). There was no difference between newly induced T_reg_ in the spleens of PLGA‐ATRA MP‐treated mice and PLGA‐Blank MP‐treated mice (Figure 7j).

### 2.7 PLGA‐ATRA MP Treatment Is Effective without Generalized Immunosuppression

**Figure 8:**
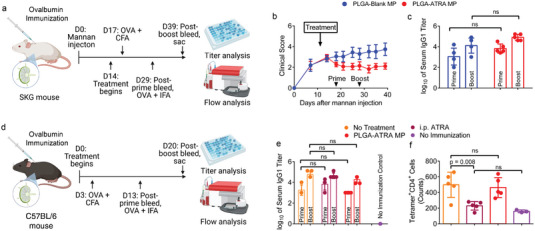
IA PLGA‐ATRA MP do not impair systemic immune response against an arthritis‐irrelevant antigen. a) Experimental schematic for ovalbumin (OVA) immunization in arthritic SKG mice. b) Clinical scores of SKG mice treated with either IA PLGA‐Blank MP (*n* = 5) or PLGA‐ATRA MP (*n* = 6). c) Anti‐OVA serum IgG1 titers before a booster immunization and 10 days after booster immunization. d) Experimental schematic for OVA immunization in nonarthritic B6 mice. e) Anti‐OVA serum IgG1 titers and f) QAVHAAHAEIN Tetramer^+^CD4^+^ T cell counts from mouse splenocytes 20 days after initial immunization with OVA. Data in (b) represent mean ± SEM for the PLGA‐Blank MP (*n* = 5) and PLGA‐ATRA MP (*n* = 6) groups; data in (c, e, f) represent mean ± SD of *n* = 5 per group. Statistical analysis in (b) was performed by linear mixed effects model analysis; statistical analyses in (c, e, f) were performed using one‐way ANOVA with post hoc Tukey test. Schematics in (a) and (d) were composed using BioRender.

To assess if the T_reg_ enhancement induced by IA PLGA‐ATRA MP results in generalized suppression of T cell‐mediated responses, the response of arthritic SKG mice, treated with either PLGA‐Blank MP or PLGA‐ATRA MP, was measured after prime/boost immunization. The primary immunization consisted of subcutaneous injection of an emulsion of ovalbumin (OVA), an SKG arthritis‐irrelevant antigen, in complete Freund's adjuvant 3 days post‐IA injection, followed by a booster immunization 10 days later consisting of OVA in incomplete Freund's adjuvant (**Figure** **8**a). As this route of OVA immunization is known to produce a strong anti‐OVA IgG1 antibody response, the post‐prime and post‐boost anti‐OVA IgG1 antibody concentration in the peripheral blood was quantified. Healthy nonimmunized SKG mice without arthritis were used to quantify the baseline immune response. Arthritis progression, as assessed by clinical scoring, was not affected by either the prime or boost immunization in both PLGA‐Blank MP and PLGA‐ATRA MP mice and was similar to nonimmunized mice (Figure 8b). Plasma anti‐OVA IgG1 antibody titers were comparable between PLGA‐Blank MP‐ and PLGA‐ATRA MP‐treated mice, and both groups produced high antibody titers (Figure 8c).

To quantify the effect of IA PLGA‐ATRA MP on arthritis‐irrelevant T cell suppression, OVA‐specific tetramers for quantifying antigen‐specific T cells in H2^b^‐background mice were used and the aforementioned immunization study in healthy C57BL/6J (B6) mice was conducted (Figure 8d). Systemically administered ATRA, delivered as a daily intraperitoneal (i.p.) injection, was also used to assess the effect of systemic exposure. The anti‐OVA IgG1 titer in IA PLGA‐ATRA MP‐treated mice was comparable to that in immunized mice that received no treatment and in mice receiving daily ATRA injections (Figure 8e). OVA‐specific CD4^+^ T cells, as quantified by I‐A(b) QAVHAAHAEIN tetramer staining, were significantly lower after daily i.p. administration of ATRA in the spleen, while a single dose of IA‐injected PLGA‐ATRA MP did not impair the antigen‐specific CD4^+^ T cell response relative to untreated immunized mice (Figure 8f).

### 2.8 PLGA‐ATRA MP Treatment Modulates Disease in the Mouse Model of CIA

**Figure 9:**
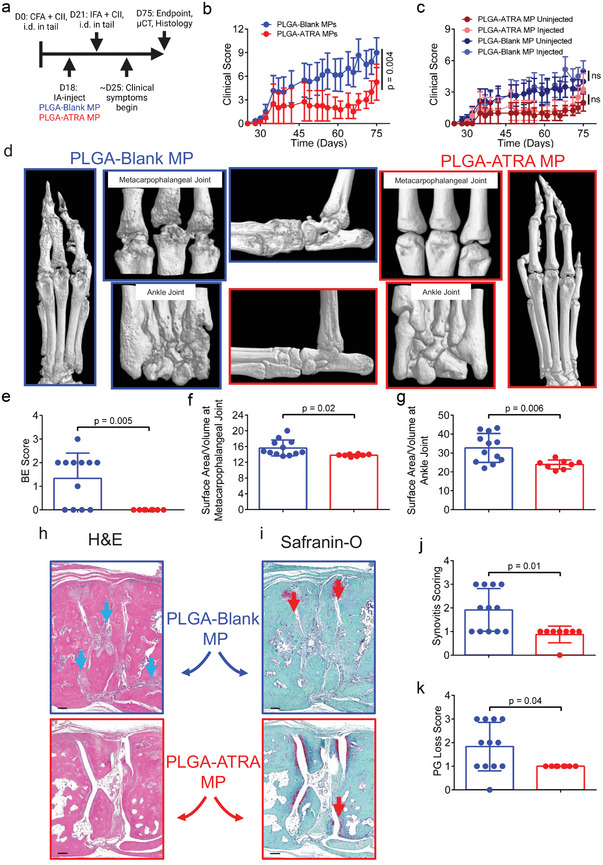
IA PLGA‐ATRA MP reduce synovitis, cartilage degradation, and bone erosions in the CIA mouse model. a) Experimental timeline for CIA induction and treatment. b) Aggregated clinical scores and c) disaggregated by injected versus uninjected joints of CIA mice treated with a 2 µg of either IA PLGA‐Blank MP (*n* = 6) or IA PLGA‐ATRA MP (*n* = 5) in both hind paws (total dose 4 µg). d) Representative micro‐CT images of hind paws from PLGA‐Blank MP and PLGA‐ATRA MP treated joints. Magnified images were used for treatment‐blinded e) bone erosion and f,g) bone surface area to volume quantification for the f) metacarpophalangeal and g) ankle joints. Representative h) H&E‐ and i) safranin O‐stained histological ankle sections from mice that received either PLGA‐Blank MP or PLGA‐ATRA MP depicting synovitis (green arrows) and proteoglycan loss (red arrows). Treatment‐blinded j) synovitis and k) proteoglycan loss scores from H&E and safranin O histological sections, respectively. Data represent b,c) mean ± SEM or e–g,j,k) mean ± SD. Statistical analysis was performed using b,c) linear mixed effects model analysis, e,j,k) Mann–Whitney test, or f,g) Student's *t*‐test, h,i) scale bar: 100 µm. Schematic in (a) was composed using BioRender.

To further validate the efficacy of PLGA‐ATRA MP, the disease modifying effect was assessed in the CIA mouse model. DBA/1 mice were primed with an emulsion of complete Freund's adjuvant and collagen and subsequently treated with either 2 µg of PLGA‐ATRA MP or PLGA‐Blank MP in both ankles via IA injection (2x total dose used in the SKG mouse model) 3 days before boost, prior to the manifestation of clinical symptoms (**Figure** **9**a). Clinical scores in injected (hind) and uninjected (fore) joints of PLGA‐ATRA MP‐treated mice were significantly diminished for ≈6 weeks post‐IA injection compared to PLGA‐Blank MP‐treated mice, prior to a subsequent increase (Figure 9b,c). Analysis of high‐resolution micro‐computed tomography (CT) images of the metacarpophalangeal and ankle joints confirmed strong protection against bone erosions by PLGA‐ATRA MP as assessed by BE scoring of CT images and significantly lower bone surface area to volume ratio compared to the PLGA‐Blank MP‐treated mice (Figure 9d–g). Ankles were subsequently processed for histomorphometry (Figure 9h,i). Scoring of H&E‐ and safranin‐O‐stained sections of the navicular cuneiform joint confirmed reduced synovitis and PG loss, respectively, in PLGA‐ATRA MP‐treated mice compared to PLGA‐Blank MP treatment (Figure 9j,k).

## 3 Discussion

Here, we demonstrate that a single IA injection of PLGA‐ATRA MP systemically reduced inflammation severity in arthritic SKG mice, which correlated with enhancing anti‐inflammatory T_reg_ over proinflammatory Th17 cells. We confirmed that ATRA promoted the differentiation of mouse and human T_reg_ and inhibited Th17, consistent with prior reports, and prevented mouse T_reg_ destabilization.^[^ ^42^, ^43^, ^44^ ^]^ The immunophenotypic modulation was consistent with ATRA‐mediated enhanced chromatin accessibility at T_reg_‐relevant loci and reduced that of Th17‐associated loci in Th17 polarizing conditions. A single bolus IA injection of ATRA had no effect on arthritis progression and encapsulation of ATRA in PLGA‐based microparticles facilitating sustained release of ATRA at therapeutically relevant concentrations was necessary to suppress disease progression for at least 4 weeks post‐injection. Importantly, PLGA‐ATRA MP, in addition to reducing inflammation, also acts as a systemic disease‐modifying agent in the SKG and CIA mouse models of arthritis. The IA‐based immunoregulatory approach is distinct conceptually from IA steroid injections which are used in some RA patients but are neither disease modifying nor are known to cause benefit to uninjected joints.^[^ ^45^ ^]^ The therapeutic efficacy of PLGA‐ATRA MP also compares favorably to systemically administered DMARDs in SKG and CIA mice. The improvement in arthritis scores by IA PLGA‐ATRA MP is superior to the reported efficacy of methotrexate in SKG mice, a first line DMARD, even though it is administered at a dose (1 mg kg^−1^ daily) which is ≈20‐fold greater than the typical clinically used dose of methotrexate in humans.^[^ ^46^ ^]^ The efficacy of IA PLGA‐ATRA MP was comparable to weekly systemic administration of 100 µg anti‐IL‐17A antibody treatment in SKG mice or weekly systemic administration of 200 µg anti‐IL‐6R.^[^ ^22^ ^]^

Although no mouse model of RA entirely recapitulates the human disease, pre‐arthritic and arthritic SKG mice are good models for understanding key pathophysiological features of human RA.^[^ ^38^ ^]^ Importantly, the SKG mouse model of RA recapitulates well the insufficient T_reg_ function and stability described in many patients with RA.^[^ ^47^, ^48^ ^]^ We had previously demonstrated that SKG T_reg_ can downregulate FoxP3 and convert to pathogenic IL‐17A^+^ ex‐T_reg_ in arthritic joints and draining lymph nodes.^[^ ^22^, ^41^ ^]^ Inspired by these findings, here we demonstrated that ATRA enhanced differentiation of naïve CD4^+^ T cells into T_reg_ over Th17 in Th17 polarizing conditions and enhanced stability of pre‐existing T_reg_, which is consistent with prior observations of ATRA on T_reg_.^[^ ^49^, ^50^, ^51^, ^52^, ^53^, ^54^, ^55^, ^56^ ^]^ Using ATAC‐Seq analysis, we correlated the immunophenotypic modulation to differential changes mediated by ATRA in the chromatin landscape which correlated with differences in H3K4me3 methylation at the *Foxp3* locus. These observations are consistent with the known effect of ATRA on enhancing accessibility of transcription machinery to the *Foxp3* promoter via histone methylation.^[^ ^52^, ^57^ ^]^ Even though ATAC‐Seq analysis supported that Th17‐associated loci were differentially accessible, the similarity in H3K4me3 methylation at Th17‐associated loci suggests that this ATRA‐mediated epigenetic modification did not directly contribute to changes in chromatin accessibility at these sites. Here, the observation that Runt family transcription factors are enhanced by ATRA is notable as it has been previously shown that these factors are important in facilitating the heritable maintenance of the active state of the *Foxp3* locus.^[^ ^30^, ^42^ ^]^ Moreover, the finding that ATRA stabilizes pre‐existing natural (n)T_reg_ is consistent with prior work that has demonstrated that ATRA can directly inhibit the methylation of the *Foxp3* gene and maintains accessibility in the presence of inflammatory cytokines.^[^ ^58^, ^59^ ^]^ The composition, size, and stability of nT_reg_ are controlled by noncoding DNA sequence (CNS) elements at the *Foxp3* locus. ATRA has also been demonstrated to contribute to nT_reg_ stability by preventing demethylation of the FoxP3 enhancer CNS2.^[^ ^59^ ^]^ However, demethylation at the CNS2 locus of induced T_reg_ (iT_reg_) has been demonstrated to be unaffected, regardless of ATRA treatment.^[^ ^52^ ^]^ Consistent with this observation, T_reg_‐ and Th17‐associated loci did not exhibit differential CpG methylation patterns between cells treated with and without ATRA. Taken together, the data support that ATRA distinctly promotes iT_reg_ induction and nT_reg_ stability.

In addition to enhancing T_reg_, we show that ATRA modulates the phenotypes of DCs and macrophages, which are myeloid cell subsets. ATRA reduced the expression of the DC costimulatory markers CD80 and CD86 and TNF expression in stimulated macrophages. DCs have been shown to process and present self‐antigens to prime autoimmune responses in lymphoid organs, such as the draining lymph node, which leads to inflammation in the joint.^[^ ^60^, ^61^, ^62^, ^63^, ^64^ ^]^ In vivo, PLGA‐ATRA MP were taken up by myeloid cells in the injected but not uninjected joint. Thus, local immunomodulation of DCs could reduce local joint inflammation and may also facilitate the induction of disease protective T_reg_ that recirculate. The effect of ATRA‐mediated reduction in the inflammatory macrophages is consistent with prior reports.^[^ ^55^, ^65^, ^66^ ^]^ However, macrophages have limited capacity for recirculation, though some subsets could act as antigen presenting cells in the draining lymph node.^[^ ^67^ ^]^ As PLGA‐ATRA MP uptake is restricted to myeloid cells in the injected joint, the resulting immunomodulatory effect is unlikely to directly contribute to systemic disease modulation through recirculation of DCs and macrophages.

IA PLGA‐ATRA MP are distinct from other preclinical immunoregulatory strategies in autoimmune inflammatory arthritis that are being explored as alternatives to generalized immunosuppression such as localized delivery of engineered DMARDs, antigen‐specific immunomodulation using tolerogenic vaccines in CIA mice, ex vivo generation and infusion of tolerogenic T_reg_‐inducing dendritic cells by incubation with a mixture of RA autoantigens (Rheumavax), ex vivo generation and infusion of a chimeric antigen receptor (CAR)‐T_reg_ against one or multiple RA autoantigens.^[^ ^68^, ^69^, ^70^, ^71^, ^72^, ^73^ ^]^ While all these strategies are effective and translatable to the clinic, the efficacy of Rheumavax and CAR‐T_reg_ approaches could be limited by the paucity of known autoantigens that can be targeted as substantial antigenic spread is known to occur in RA. A localized immunosuppression approach has also been explored through IA administration of anti‐TNF Ab, modified with an extracellular matrix‐binding peptide. While the IA agent suppressed arthritis in treated joints, it did not display systemic efficacy.^[^ ^72^ ^]^ PLGA‐ATRA MP have the advantage of acting locally at the site of inflammation to promote disease‐specific immunomodulation without a priori knowledge of participating epitopes. Other methods using biomaterial depots to expand T_reg_ have used PLGA microparticles encapsulating multiple immunoregulatory agents, such as rapamycin, IL‐2, and TGF‐*β* that, when injected near hind paw draining lymph nodes, modulated disease but also resulted in a systemic immunosuppression and nonspecific expansion of T_reg_.^[^ ^74^, ^75^ ^]^ Moreover, PLGA‐ATRA MP can be lyophilized and stored for extended periods and potentially used off‐the‐shelf and similar to other IA‐injected agents, if needed, PLGA‐ATRA MP injections could be performed under fluoroscopic and ultrasound guidance techniques. Importantly, systemically delivered ATRA is not associated with musculoskeletal symptoms, further supporting the idea that ATRA will be well‐tolerated and safe for IA delivery.^[^ ^53^, ^76^ ^]^

PLGA‐ATRA MP equally protected injected ipsilateral and uninjected contralateral joints, and systemic disease modulation was not associated with generalized suppression of T cell‐dependent immune responses. The ex vivo ability to induce and stabilize T_reg_, combined with the reduction of Th17 cells in the ankles and draining lymph nodes of PLGA‐ATRA MP‐treated SKG mice and the similar findings obtained by single IA injection of T_reg_ in the same model support that local differentiation/stabilization of T_reg_ followed by T_reg_ systemic recirculation underlies the systemic improvement in arthritis progression observed after a single IA injection of PLGA‐ATRA MP.^[^ ^77^ ^]^ An enhanced fraction of newly induced T_reg_ in the joints of mice treated with PLGA‐ATRA MP, distinct from the subset that is stabilized, may also contribute to improved control of inflammation, which is consistent with prior work that has demonstrated the role of induced T_reg_, which have been show to retain their phenotype and function in cytokine‐driven autoimmune arthritis.^[^ ^78^ ^]^

In inflamed RA joints, capillary permeability increases to allow cell migration, an effect that also enhances the exit of molecules, especially low molecular weight compounds, from the joint space. Therefore, although bolus IA injection of ATRA can localize the effect of ATRA, it cannot avoid rapid clearance. As IA injections can only be administered with limited frequency, rapid clearance cannot be overcome simply by increasing the frequency of drug administration. For this reason, amounts well above the therapeutically relevant concentration must be introduced in the joint which could cause local toxicity as well as lead to undesired side effects if taken up by off‐target tissues after exit from the joint. In contrast, PLGA‐ATRA MP enable release of local concentrations of ATRA which are at the same time sustained and effective locally but insufficient to cause systemic ATRA exposure above the immunosuppressive threshold. PLGA‐ATRA MP were effective at suppressing disease progression after a single IA injection and well‐tolerated at a dose ranging from 2 to 200 µg. The systemic exposure at these doses is predicted to be significantly lower than frequent systemically administered ATRA ranging from 0.5 to 25 mg kg^−1^ per dose, which has been used in other autoimmune disease mouse models modulating inflammation in uveitis, experimental autoimmune encephalomyelitis, and inflammatory autoimmune arthritis.^[^ ^53^, ^54^, ^55^, ^79^, ^80^ ^]^ The calculated maximum systemic exposure to ATRA in vivo after a single IA PLGA‐ATRA MP injection (*C*_max_) is 40 × 10^−12^ m, well below the concentrations reached with doses that have been associated with toxicity in rodents (>14 mg kg^−1^).^[^ ^81^, ^82^ ^]^ These results are consistent with prior work that evaluated sustained release and systemic concentration of ATRA following a subcutaneous dose.^[^ ^83^ ^]^ Moreover, our pharmacokinetic model shows that a therapeutically relevant concentration of ATRA is maintained in the synovial fluid of the treated joint and the immunization experiment demonstrates that IA released ATRA avoids generalized immune suppression.

Following IA injection of PLGA‐ATRA MP, Th17 cells were reduced in the ipsilateral ankle within 3 days. 11 days post‐treatment, reduced Th17 were observed in the ipsilateral and contralateral ankles and corresponding draining lymph nodes. These results are consistent with prior findings that have correlated disease severity with Th17 cells in SKG mice.^[^ ^22^ ^]^ Our results from fate mapping SKG mice further support that the enhancement in T_reg_ stability in the ankles and joint‐draining lymph nodes following IA injection of PLGA‐ATRA MP contribute in part to the observed reduction in Th17. Systemic recirculation of IA‐injected T_reg_ and preferential accumulation in arthritic joints over nonarthritic joints, accompanied by a corresponding decrease in clinical score support the notion that T_reg_ have the capacity to modulate arthritis severity. Furthermore, treatment of T_reg_ with ATRA enhanced the migratory marker CCR9, which has previously been associated with T cell chemotaxis occurring in a CCL25 dependent manner, a chemokine which is also commonly found in the RA joint synovial fluid.^[^ ^84^, ^85^, ^86^ ^]^ While we cannot rule out the contribution of other recirculating cells and soluble factors in mediating arthritis protection, the results from the in vitro enhancement of T_reg_ function and migratory markers mediated by ATRA, the efficacy of ATRA‐treated T_reg_ after transfer into RAG mice, the enhancement in newly induced T_reg_ in vivo by PLGA‐ATRA MP and the lack of efficacy of subcutaneously administered PLGA‐ATRA MP all support that disease‐specific immunomodulation may be attributed, at least in part, to recirculation of T_reg_ from the treated joint.

SKG arthritis symmetrically affects joints and is associated with the elevation of key RA cytokines including IL‐6, IL‐1*β*, and TNF. Moreover, joint damage manifests as cartilage and bone erosion.^[^ ^36^ ^]^ Here, we observed that PLGA‐ATRA MP‐treated mice had lower immune cell infiltration, and lower scores for BE and PG loss, in both injected and untreated joints. We also found that treatment with PLGA‐ATRA MP resulted in reductions in *Il6*, *Il1b*, *Tnf, Mmp13*, and *Mmp1* expression in both the injected and uninjected joints compared to sham injected controls, but not in *Tgfb1*. PLGA‐Blank MP alone modestly reduce these inflammatory markers in the injected ankle, but this reduction was not statistically significant. To further validate these findings of joint protection, we tested PLGA‐ATRA MP in the CIA mouse model, widely used in RA research and preclinical studies. As in SKG mice, T_reg_ in CIA mice have been shown to lose FoxP3 expression and undergo IL‐6‐mediated trans‐differentiation into exT_reg_ that accumulate in inflamed joints.^[^ ^87^ ^]^ CIA is characterized by synovial hyperplasia, immune cell infiltration, and significant bone erosions. Arthritis presentation in CIA mice is well‐known to be asymmetric and therefore both ankle joints were treated. Despite the increased dose, the model predicted that the expected systemic concentration of ATRA was well below immunosuppressive concentrations. Upon treatment, PLGA‐ATRA MP but not PLGA‐Blank MP, significantly reduced clinical scores in both the injected and uninjected joints. Treatment‐blinded analysis of high‐resolution micro‐CT images of the metacarpophalangeal joints and the bone proximal to the tibio‐tarsal junction confirmed strong protection against bone erosions by PLGA‐ATRA MP as assessed by BE scoring and a significantly lower bone surface area to volume ratio compared to the PLGA‐Blank MP‐treated mice. Treatment‐blinded histomorphometry analysis of H&E‐ and safranin‐O‐stained sections of the navicular cuneiform joint confirmed reduced synovitis and PG loss, respectively, in PLGA‐ATRA MP‐treated mice. These results further validate PLGA‐ATRA MP as an immunoregulatory agent in autoimmune arthritis.

Uncontrolled inflammatory arthritides lead to chronic pain and disability. While DMARDs have transformed disease management, a large fraction of patients face difficulty in achieving and remaining in remission. As joint damage is correlated with time to remission, clinical protocols seek to rapidly minimize disease activity. However, several patients do not respond to multiple DMARDs and in patients who are only partially responsive to specific DMARDs, deepening immunosuppression—e.g., by combining a second DMARD—increases the risk of infections and cancer. The ability to modulate pathogenic immune cells to delay clinical disease progression, coupled with preservation of cartilage PG and reduced BE, supports the utility of IA PLGA‐ATRA MP as a locally delivered agent that provides a systemic disease‐specific clinical benefit. If PLGA‐ATRA MP‐mediated immunomodulation performs similarly in the human context, then its application—especially if administered in early RA—might work as a monotherapy to stem progression of disease. More likely, one or more injections of PLGA‐ATRA MP could serve as an immunoregulatory adjuvant to treat those that are inadequately responsive or intolerant to DMARDs, or in combination with DMARDs to enhance control of disease without further impairing the immune response.

## 4 Experimental Section

### Study Design

The objective of this study was to develop an IA‐deliverable immunomodulatory agent to promote systemic disease remission in inflammatory arthritis. To this end, PLGA‐ATRA MP were formulated and characterized. The single‐emulsion method was used to generate PLGA‐ATRA MP by consistently generating at least three batches for each particle size. Subsequently, all in vitro material characterization studies were conducted at least three times. All in vitro mouse cell culture studies were performed with a minimum of three technical replicates and three experimental replicates. Three technical replicates were used for ATAC‐seq sample preparation and subsequent analysis. All in vivo studies were conducted following an approved IACUC protocol. Outcomes were generally determined by assessing clinical scores, ankle thickness, flow cytometry assessments, and histological appearances, unless otherwise noted. For in vivo arthritis studies, littermate mice were injected with mannan to synchronize disease onset. The criteria for omission were i) onset of severe arthritis in one or more paws prior to treatment, ii) signs of arthritis on day 0, and iii) failure to develop arthritis by day 14 post‐mannan injection. All other animals were included in the data analysis. Predetermined endpoints for data collection were based on changes in and progression of clinical scores in the treatment groups. Prior to treatment, littermate mice were randomly placed into treatment groups after ensuring that the mean clinical score of each group was the same. All arthritis studies were conducted in at least two litters of mice. For fate mapping studies, littermate mice were randomly placed into treatment groups prior to mannan injection. For ovalbumin immunization studies, cages that received treatment prior to ovalbumin immunization were randomly chosen. In general, statistical power for arthritis studies was based on prior reports. Sample numbers for each individual experiment are provided in the figure legends. Alphanumeric coding was used to blind treatment groups for flow cytometry and histomorphometry analysis. Scoring was performed by two independent operators.

### Materials

PLGA (50:50, AP041, lot: 200825RAI‐B, MW 10–15 kDa) and Cy5‐labeled PLGA (50:50 PLGA‐Cy5, AV034, lot: 201215RAI‐A, MW 10–15 kDa) were purchased from Akina. All‐trans retinoic acid (BML‐GR100‐0500, lot: 06011830) was purchased from Enzo. Dimethyl sulfoxide (DMSO, D128‐500, lot: 194474) and dichloromethane (DCM, D143‐1, lot: 194105) were purchased from Fischer Chemical. Poly‐vinyl alcohol (363146‐500G, lot: MKCF9787, MW 85–124 kDa), corn oil (C8267‐500ML, lot: MKCM9808), and mannan (M7504‐5G, lot: SLCF4977) were purchased from Sigma‐Aldrich. Roswell Park Memorial Institute (RPMI) powder was purchased from (Gibco, lot: 2344357). Mannan was purchased from Sigma‐Aldrich. Incomplete Freund's Adjuvant + Ovalbumin (EK‐0311, lots: 105, 111) and Complete Freund's Adjuvant + Ovalbumin (EK‐0301, lots: 105, 111) were purchased from Hooke Labs.

### In Vitro Mouse T Cell Differentiation Assays

Naïve SKG mouse CD4^+^ T cells were isolated via magnetic depletion using the Naïve CD4^+^ T cell Isolation kit (Miltenyi Biotech) according to the manufacturer's instructions. Isolated naïve CD4^+^ T cells were plated in 96‐well plates at 100 000 cells per well in RPMI 1640 supplemented with nonessential amino acids (NEAA), sodium pyruvate, and beta‐mercaptoethanol (complete RPMI). To induce Th17 differentiation, IL‐6 (50 ng mL^−1^) (200‐06, lot: 031916‐1 B2321), TGF‐*β*1 (5 ng mL^−1^) (100‐21, lot: 1218209 H1919), IL‐23 (5 ng mL^−1^) (200‐23, lot: 0712S227 K1119), and IL‐1*β* (20 ng mL^−1^) (200‐01B, lot: 0606B95 B2421) were added at the time of plating, with various concentrations of ATRA. All cytokines were recombinant human cytokines purchased from Peprotech. Cells were stimulated with phorbol myristic acetate (PMA)‐ionomycin (Cell Stimulation Cocktail, Invitrogen, Cat. No. 00‐4970‐03, lot: 2430454) and brefeldin A (Invitrogen, Cat. No. 00‐4506‐51) for 5 h prior to staining and analysis.

For the ATRA pretreatment Th17 differentiation assay, the same isolation procedure was followed. Immediately following isolation, cells were plated at 100 000 cells per well in complete RPMI with Dynabeads to provide stimulation at a concentration according to the manufacturer's instructions. Media was supplemented with or without 1 × 10^−9^ m ATRA. Following either 24 or 48 h of incubation at 37 °C, the cells were washed four times using a 1:10 dilution to ensure ATRA concentration in the media was below 1 × 10^−12^ m. The cells were then resuspended and transferred to a new plate and RPMI with Th17 inducing cytokines as detailed previously were added, and cells were incubated for an additional 72 h before being stimulated with PMA‐ionomycin and brefeldin A for 5 h prior to staining and analysis.

For the ATRA post‐treatment following Th17 polarization assay, the same isolation procedure was followed. Isolated naïve CD4^+^ T cells were plated in 96‐well plates at 100 000 cells per well in complete RPMI with Th17 differentiating factors as described above. After 5 days, cells were analyzed and split into a group that received 1 × 10^−9^ m ATRA and a control group that did not receive 1 × 10^−9^ m ATRA for 48 h. Cells were then stimulated with PMA‐ionomycin and brefeldin A for 5 h prior to staining and analysis.

For the generation of +ATRA T_reg_ for transfer, cells were isolated as previously described. Naïve CD4^+^ T cells were then plated at 100 000 cells per well in 200 µL of complete RPMI with Dynabeads to provide stimulation according to manufacturer's instructions. Complete RPMI media was supplemented with 1 × 10^−9^ m ATRA, 5 ng mL^−1^ IL‐2, and 5 ng mL^−1^ TGF‐*β*. Cells were cultured for 7 days, with 300 µL of media addition and transfer to a 24‐well plate on day 3 and 500 µL media addition on day 5. Added media contained all factors listed.

### In Vitro Mouse T_reg_ Destabilization Assay

Total FoxP3^eGFP+^TCR*β* ^+^CD4^+^ T cells were sorted using Fluorescence‐Activated Cell Sorting (FACS) using a Sony SH800S Cell Sorter (SH800S) from the spleens and lymph nodes of 8–12 weeks old female BALB/c FoxP3^eGFP^ SKG mice or 8–12 weeks old C57BL/6J FoxP3^eGFP^ SKG fate‐mapping mice. After sorting, FoxP3^+^ T_reg_ were stimulated using plate‐bound *α*CD3 and *α*CD28 (adsorbed at 5 µg mL^−1^ in PBS at 37 °C for 3 h) with or without IL‐6 (Peprotech) at 50 ng mL^−1^ in the presence of varying concentrations of DMSO‐solubilized ATRA or PLGA‐ATRA MP. After 72 h, cells were removed, washed, and stimulated with PMA‐ionomycin and brefeldin A for 5 h and analyzed for expression of FoxP3, IL‐17A, and ROR*γ*t via flow cytometry. Results from assays that yielded an overall cell viability of less than 50% at the endpoint were excluded.

### In Vitro Human Th17 Differentiation Assay

Fresh whole blood was purchased from the La Jolla Institute for Allergy and Immunology (LJI) and used fresh on the day of the draw. Peripheral blood mononuclear cells (PBMCs) were isolated using a Ficoll density gradient (Lymphopure). Isolated PBMCs were then sorted using a Human Naïve CD4 T cell Isolation kit (Miltenyi Biotech) according to the manufacturer's instructions. Isolated naïve CD4 T cells were plated in 96‐well plates at 100 000 cells per well in RPMI 1640 supplemented with NEAA, sodium pyruvate, and beta‐mercaptoethanol. To induce Th17 differentiation, IL‐6 (50 ng mL^−1^), TGF‐*β*1 (5 ng mL^−1^), IL‐23 (5 ng mL^−1^) and IL‐1*β* (20 ng mL^−1^), and IL‐21 (200‐21, 1019226) (25 ng mL^−1^) were added at the time of plating, with various concentrations of ATRA. All cytokines were recombinant human cytokines purchased from Peprotech and were the same as those used in mouse T cell differentiation assays, with the exception of IL‐21.

### In Vitro Dendritic Cell Differentiation and Stimulation Assays

BMCs were harvested from the tibia and femurs of SKG mice. Following red blood cell lysis, cells were resuspended in complete Dulbecco's modified Eagle medium (cDMEM) which contained DMEM prepared according to manufacturer's instructions (12100‐046, Gibco) as well as 1% nonessential amino acids, 1% penicillin‐streptomycin, beta‐mercaptoethanol, and 10% fetal bovine serum. For DC differentiation, 50 ng mL^−1^ GM‐CSF was added to the media and BMCs were plated in 6‐well plates with 2 mL of media at 1 million cells mL^−1^. Media was added on day 3, and whole media was refreshed on day 5. To assess the effect of ATRA on DC differentiation, 10 × 10^−9^ m ATRA was added to select wells on day 0 and the concentration was kept constant when adding and replacing media. To stimulate DCs, 10 ng mL^−1^ LPS was added on day 7. Brefeldin A was added on day 8 for 4 h prior to harvesting cells via trypsinization for staining and flow cytometry.

### In Vitro Macrophage Differentiation and Stimulation Assays

BMCs were harvested from the tibia and femurs of SKG mice. Following red blood cell lysis, cells were resuspended in cDMEM. For macrophage differentiation, 25 ng mL^−1^ M‐CSF was added to the media and BMCs were plated in 6‐well plates with 2 mL of media at 1 million cells mL^−1^. Media was added on day 3, and whole media was refreshed on day 5. To assess the effect of ATRA on macrophage differentiation, 10 × 10^−9^ m ATRA was added to select wells on day 0 and the concentration was kept constant when adding and replacing media. To stimulate and polarize macrophages, 10 ng mL^−1^ LPS and 50 ng mL^−1^ IFN*γ* were added on day 7. Brefeldin A was added on day 8 for 4 h prior to harvesting cells via trypsinization for staining and flow cytometry.

### ATAC‐Seq Library Preparation and Sequencing

Cells used in ATAC‐Seq experiments were subjected to the same procedure as described in Section *In Vitro Mouse Th17 Differentiation Assay*. On day 3, 10^5^ cells were removed from media and prepared for ATAC‐Seq using the Active Motif ATAC‐Seq kit (Active Motif, Cat No. 53150) according to manufacturer's instructions. Briefly, isolated cells were washed once with ice‐cold PBS and subsequently resuspended in ATAC lysis buffer on ice to isolate the nuclei. The lysis buffer was removed, cells were washed, and nuclei were incubated with tagmentation mix at 37 °C for 30 min, after which DNA was isolated and purified. DNA was then amplified via PCR using the provided indexed primers according to manufacturer's instructions. SPRI cleanup beads were then used to purify the amplified DNA prior to sequencing. Samples were sequenced at the La Jolla Institute for Allergy and Immunology using an Illumina NovaSeq sequencer (NovaSeq 6000).

### ATAC‐Seq Analysis

Analysis of raw reads from sequencing of ATAC‐seq prepared libraries went as follows: FASTQ files were aligned to mm10 reference genome using bowtie2 with parameters “‐k 4 ‐X 2000.”^[^ ^88^ ^]^ Reads corresponding to Encyclopedia of DNA Elements (ENCODE) blacklisted regions, mitochondrial genome, and chrY were removed.^[^ ^89^ ^]^ Reads were then filtered for uniquely mappable reads with mapping quality > = 30 using SAMtools, and duplicate reads were removed using MarkDuplicates from Picard tools (https://broadinstitute.github.io/picard).^[^ ^90^ ^]^ Reads aligning to the (+) strand were shifted by +4 bp and reads aligning to the (−) strand were shifted by −5 bp using deepTools alignmentSieve.^[^ ^91^ ^]^ Peaks were then called using MACS2 with the parameters (*‐g mm ‐q 0.05 –nomodel –nolambda –keep‐dup all –call‐summits –shift ‐100 –extsize 200*).^[^ ^92^ ^]^ Summits of all peaks were then extended to regions of 500 bp, and the 500 bp peaks from +ATRA group and −ATRA group were merged to get consensus peaks via bedtools slop and merge routines. Shifted BAM files were then converted to beds and the reads for each region of the consensus peaks were counted using bedtools coverage.^[^ ^93^ ^]^ Count normalization and differential ATAC‐seq analysis was then performed using DESeq2.^[^ ^94^ ^]^ DARs were filtered using FDR‐adjusted *p*‐values < = 0.05 and fold‐change > = 2. Normalized coverage tracks were created from shifted BAM filesusing deepTools bamCoverage with parameters “‐bs 10 –effectiveGenomeSize 2652783500 –normalizeUsing RPKM ‐e 200.”^[^ ^91^ ^]^ Motif enrichment analysis was performed by clustering the DARs into two clusters using k‐means. Hypergeometric Optimization of Motif EnRichment (HOMER) was then used to identify known motifs for transcription factor binding sites enriched in the different clusters of peaks.^[^ ^95^ ^]^

### CUT‐and‐TAG Library Preparation and Sequencing

Cells used in CUT‐and‐TAG experiments were subjected to the same procedure as described in Section *In Vitro Mouse Th17 Differentiation Assay*. On day 3, 1x10^5^ cells were removed from media and cryopreserved. Cryopreserved mouse primary CD4^+^ T cells were sent to Active Motif for the CUT&Tag assay. Cells were incubated overnight with Concanavalin A beads and 1 µL of the primary anti‐H3K4me3 antibody per reaction (Active Motif, catalog number 39159). After incubation with the secondary anti‐rabbit antibody (1:100), cells were washed and tagmentation was performed at 37 °C using protein‐A‐Tn5. Tagmentation was halted by the addition of ethylenediaminetetraacetic acid, sodium dodecyl sulfate, and proteinase K at 55 °C, after which DNA extraction and ethanol purification were performed, followed by PCR amplification and barcoding (see Active Motif CUT&Tag kit, catalog number 53160 for recommended conditions and indexes). Following SPRI bead cleanup (Beckman Coulter), the resulting DNA libraries were quantified and sequenced on Illumina's NextSeq 550 (8 million reads, 38 paired end).

### RRBS Preparation and Sequencing

Cells used in RRBS experiments were subjected to the same procedure as described in Section *In Vitro Mouse Th17 Differentiation Assay*. On day 3, 10^5^ cells were removed from media and cryopreserved. Cryopreserved mouse primary CD4^+^ T cells were sent to Active Motif for RRBS. Genomic DNA was extracted using the Quick‐gDNA MiniPrep kit (Zymo Research D3024) following manufacturer's instructions for cell suspensions and proteinase K digested samples. 100 ng of gDNA was digested with TaqaI (NEB R0149) at 65 °C for 2 h followed by MspI (NEB R0106) at 37 °C overnight. Following enzymatic digestion, samples were used for library generation using the Ovation RRBS Methyl‐Seq System (Tecan 0353‐32) following manufacturer's instructions. In brief, digested DNA was randomly ligated, and, following fragment end repair, bisulfite converted using the EpiTect Fast DNA Bisulfite Kit (Qiagen 59824) following the Qiagen protocol. After conversion and clean‐up, samples were amplified resuming the Ovation RRBS Methyl‐Seq System protocol for library amplification and purification.

RRBS libraries were measured using Agilent 2200 TapeStation System and quantified using the KAPA Library Quant Kit ABI Prism qPCR Mix (Roche KK4835). Libraries were sequenced on a NovaSeq 6000 at SE75.

### RRBS and CUT‐and‐TAG Analysis

Reads were aligned using the BWA algorithm (mem mode; default settings). Duplicate reads were removed, and only reads that mapped uniquely (mapping quality > = 1) and as matched pairs were used for further analysis. Alignments were extended in silico at their 3’‐ends to a length of 200 bp and assigned to 32‐nt bins along the genome. The resulting histograms (genomic “signal maps”) were stored in bigWig files. Peaks were identified using the MACS 2.1.0 algorithm at a cutoff of *p*‐value 1e‐7, without control file, and with the –nomodel option. Peaks that were on the ENCODE blacklist of known false ChIP‐Seq peaks were removed. Signal maps and peak locations were used as input data to Active Motifs proprietary analysis program, which created Excel tables containing detailed information on sample comparison, peak metrics, peak locations, and gene annotations. For differential analysis, reads were counted in all merged peak regions (using Subread), and the replicates for each condition were compared using DESeq2. Fold change between +ATRA and −ATRA samples was then calculated and compared, and the list of genes with an absolute value of log_2_(fold change) greater than one was generated and compared to DARs observed in ATAC‐Seq.

### Microparticle Synthesis

PLGA‐Blank and PLGA‐ATRA MP were synthesized via an oil‐in‐water emulsion. PLGA was dissolved at 50 mg mL^−1^ in DCM. For fluorescence, 1 mg mL^−1^ of PLGA‐Cy5 was included. For preparing PLGA‐ATRA MP, 100 µL of 20 mg mL^−1^ ATRA dissolved in DMSO was added to the PLGA solution and vortexed until fully mixed. The resulting solution was slowly dropped into 20 mL of a 2 w/v% poly(vinyl alcohol) (PVA) solution that was continuously homogenized for 1 min using a handheld homogenizer (Benchmark Scientific D1000 Handheld Homogenizer) to create the emulsion. PLGA‐Blank MP and PLGA‐ATRA MP were prepared at homogenization speeds of 8500, 17 100, and 30 000 rpm with a 0.007 m impeller. The primary emulsion was then added to 60 mL of 1 wt% PVA stirring at 400 rpm and left stirring for 4 h at room temperature. The resulting mixture was then centrifuged at 1000 *g* and washed using sterile deionized water four times before it was frozen and lyophilized. The resulting size distribution was analyzed using scanning electron micrographs (SEMs).

### Scanning Electron Micrographs

Samples of lyophilized microparticles were prepared as follows: lyophilized microparticles stored at −20 °C were mechanically agitated until the product was freely moving powder. A small amount of power was placed on carbon tape affixed to an SEM stub, and excess powder was removed by blowing compressed air over the surface. Prepared stubs were sputter coated in an Emitech K575X Sputter Coater under argon at 4 × 10^−3^ mbar using iridium deposited using 85 mA of current for 8 s. Coated samples were then loaded into the scanning electron microscope (FEI Quanta FEG 250) and imaged using a 3 kV beam in high vacuum. Images were taken after repeated rounds of focusing and direct adjustments were performed to ensure image quality.

### Microparticle Size Distribution Analysis

The size distribution of PLGA MP was analyzed as outlined graphically in Figure S2a in the Supporting Information. Briefly, SEM images of PLGA‐ATRA MP and PLGA‐Blank MP were loaded into the FIJI distribution of ImageJ. The scales of images were set using the “Set Scale” function in conjunction with the SEM scale bar, which was subsequently cropped out. Particle edges were detected using the “Find Edges” function, and the image was converted to a binary mask using the “Convert to Mask” function. The holes in the image were filled using the “Fill Holes” function, and then the particle edges were eroded to allow for detection of distinct particles using the “Erode” function. This decreased the particle size by a set diameter, which was measured and accounted for in subsequent analysis. The particle sizes were then analyzed using the “Analyze Particles” function, and the resulting areas were converted to diameters, the diameters were adjusted to account for loss during the erosion function, and the volume‐averaged size distribution was calculated. The results of this technique were validated by comparison to hand measurement results using select images and were found to be in close agreement.

### In Vitro Release and Degradation Assay

To quantify the rate of ATRA release from PLGA‐ATRA MP, particles were suspended at 10 mg mL^−1^ in PBS with 0.1 wt% BSA and placed suspended particles in an incubator at 37 °C. To collect the supernatant to measure ATRA release, PLGA‐ATRA MP were spun down at 1000*g* for 5 min, and the supernatant was carefully collected and refreshed without disrupting the particle pellet. After the supernatant was exchanged, particles were resuspended via vigorous vortexing, and the particles were placed back into the 37 °C incubator. The concentration of ATRA in the collected supernatant was determined using a Nanodrop UV‐vis spectrophotometer via comparison to a standard curve. The release buffer was replaced every day for the first 4 days and subsequently on days 7, 14, 21, 28.

### PK Modeling

Theoretical in vivo pharmacokinetics were determined using a two‐compartment model as illustrated in Figure S3 in the Supporting Information. A concise explanation of two‐compartment pharmacokinetic models is available elsewhere.^[^ ^96^ ^]^ Here, the two‐compartment model was used to represent the synovial compartment and the peripheral blood. The governing equation was modified for the synovial compartment to include a term to account for the release of ATRA from the IA‐injected PLGA‐ATRA MP. The rate of ATRA release from PLGA‐ATRA MP was determined using the vitro release profile. The rate of exchange between the periphery and the synovium was an order of magnitude approximation based on prior reports. The rate of elimination of ATRA from the peripheral blood was based on reported half‐life of ATRA in the serum. The model was programmed and implemented in Python using the Anaconda 3 distribution and is publicly available at https://github.com/Shah‐Lab‐UCSD/PLGA‐ATRA‐PK.

### Arthritis Models

All animal work was approved by the University of California, San Diego (UCSD) Institutional Animal Care and Use Committee (IACUC) under protocol # S17160. C57BL/6J (B6, Jax # 000664), BALB/cJ (BALB/cJ, Jax # 000651), DBA/1 (Taconic # DBA1BO‐F), and BALB/c RAG2‐KO (Taconic # 601) were purchased. BALB/c SKG mice were obtained through a Materials Transfer Agreement between UC San Diego and Kyoto University and colonies were maintained at UCSD. B6.H2d.FoxP3^EGFP.ERT2‐Cre^. R26^tdTomfl/fl^.SKG mice were previously described^[^ ^47^ ^]^ and colonies were maintained at UCSD. BALB/c SKG, BALB/cJ, DBA/1, and RAG2‐KO mice used were female. B6.H2d.FoxP3^EGFP.ERT2‐Cre^. R26^tdTomfl/fl^.SKG mice were both male and female. All mice were housed under specific pathogen‐free conditions. Arthritis onset was induced in 8–12 weeks old mice via mannan injection as described elsewhere.^[^ ^47^ ^]^ Mice were injected intraperitoneally with 20 mg of mannan (Millipore‐Sigma, M7504‐5G, lot: SLCJ9322) dissolved in 200 µL of sterile PBS. Mice were anesthetized using isoflurane and disease severity was determined twice weekly using clinical scoring and measurement of hind paw swelling using calipers in accordance with a standardized protocol. Briefly, fore and hind paws were assessed independently in each mouse and were assigned scores according to the following criterion: No visible swelling (0), mild to moderate swelling (0.5), severely swollen (1.0), as well as an additional 0.1 for each swollen digit. Clinical scores reported were the aggregate of all paws (maximum of 5.8) from a single mouse unless otherwise noted. A score of 5.5 was considered the clinical endpoint and mice that attained this score before the end of the study were sacrificed according to IACUC guidelines.

To assess the efficacy of PLGA‐ATRA MP, arthritis was induced as described above. On day 14 after arthritis induction, littermate mice received an injection of either 2, 20, or 200 µg PLGA‐ATRA MP, 200 µg Blank MP suspended in 20 µL sterile PBS, or dose‐matched bolus ATRA suspended in 20 µL sterile corn oil, injected IA into a single hind ankle. The contralateral hind ankle was injected with PBS or Cy5‐labeled PLGA‐Blank MP as a sham control. Clinical scores and ankle thickness of mice were tracked bi‐weekly for an additional 3–21 days after treatment to an endpoint of 35 days after mannan injection. To examine immunological changes after injection of the PLGA‐ATRA MP, some cohorts of mice were sacrificed on day 17 after mannan injection (3 days after treatment) and some were sacrificed on day 25 after mannan injection (11 days after treatment). To ensure that all mice were at the same stage of arthritis onset, mice that developed severe arthritis (clinical score of 4.0 or greater or a minimum of 1 paw severely swollen) prior to day 14 were excluded from the study. Mice that did not manifest arthritis were excluded from the study.

For inducing arthritis in RAG2‐KO mice, CD4^+^CD25^−^ T cells were sorted from SKG lymph nodes and spleens and transferred to RAG2‐KO mice via retro‐orbital injection. 7 days after the transfer of the CD4^+^CD25^−^ T cells, RAG2‐KO mice were injected with 200 µg of mannan to initiate arthritis onset. 11 days after mannan injection, mice were treated via IA sham injection of PBS or 3x10^6^ ex vivo generated T_reg_ treated with ATRA, as described above. Clinical scores and ankle thickness were tracked following the initial cell transfer, and mice were scored bi‐weekly using the same scoring system as SKG mice.

For the CIA model, 8 weeks old female DBA/1 mice were injected with 50 µL of a complete Freund's adjuvant/bovine collagen emulsion (EK‐0220, lot 124, Hooke Laboratories). On day 18, mice received IA injection of 2 µg of either PLGA‐ATRA MP or PLGA‐Blank MP in both hind ankles. On day 21, all mice received 50 µL of incomplete Freund's adjuvant/bovine collagen emulsion (EK‐0221, lot 124, Hooke Laboratories). Fore paws and hind paws were assessed for clinical signs of arthritis according to the following criteria: No swelling or erythema (0), mild localized swelling or erythema (1), moderate localized swelling or erythema (2), moderate whole paw swelling or erythema (3), severe whole paw swelling or erythema (4). Criteria for euthanasia were a clinical score of 14 or higher.

### In Vivo Particle Uptake, Tracking, and Degradation Assays

Cy5‐labeled PLGA was incorporated into PLGA‐ATRA MP and PLGA‐Blank MP as described under synthesis to create fluorescently labeled MP. For degradation studies, arthritic mice were IA injected with 200 µg fluo‐MP and fluorescent signal was tracked bi‐weekly using an IVIS (Xenogen). Mice were anesthetized and imaged in a prone position with the site of injection oriented upward. The radiant efficiency was measured and normalized to the highest radiant efficiency value measured in the first 3 days. For particle uptake and biodistribution studies, arthritic mice were injected IA with 20 µg fluo‐MP and sacrificed 1 and 5 days after injection. The kidney from the side of injection, liver, spleen, injected and uninjected ankles, and both popliteal lymph nodes were removed and fluorescent signal was quantified using IVIS. Ankles, draining lymph nodes, and spleens were dissociated and analyzed using flow cytometry.

### qPCR Preparation and Analysis

Arthritis was induced in SKG mice and was treated after 14 days with PLGA‐Blank MP and PLGA‐ATRA MP as previously described. Ankles were harvested 7 days after treatment and were flash frozen in liquid nitrogen and stored. Whole mouse joint homogenate samples were first cut and homogenized with a handheld homogenizer in TRIzol on ice (cat# 15596018 Invitrogen). RNA was purified from the chloroform phase using RNAeasy Plus Micro kit (cat# 74034 Qiagen) according to manufacturer's protocols. Sample RNA concentration was normalized, and cDNA was synthesized using the SuperScript III First‐Strand Synthesis SuperMix for real‐time quantitative reverse transcription PCR (qRT‐PCR) (cat# 11752250 Life Technologies) according to manufacturer's protocols. qPCR was performed on a Bio‐Rad CFX384 Real‐Time PCR Detection System, with Kicqstart primer assays for *Mmp13, Tgfb1, Il6, Il1b, Tnf, Mmp3*, and *Gapdh* (KSPQ12012G, Sigma) in a final concentration of 10 × 10^−6^ m and SYBR Green qPCR Master Mix (cat# 330513 Qiagen). Primer assay efficiencies were guaranteed by the manufacturer to be greater than 90%. Each reaction was measured using technical triplicates, and data were normalized to the expression levels of the housekeeping gene *Gapdh*. Results are presented as a fold‐change compared to the average expression level in the sham‐treated ankles of PLGA‐Blank‐treated mice using the ΔΔCq method.

### T_reg_ Transfer and Tracking Assay

Live TCR*β* ^+^CD4^+^FoxP3^eGFP+^ T_reg_ were isolated from SKG.FoxP3^eGFP^ mice using a Sony SH800S Cell Sorter. Sorted T_reg_ were expanded on Dynabeads (Invitrogen, 11452D, lot: 00911146) in complete RPMI supplemented with 10 ng mL^−1^ IL‐2 for 5 days according to manufacturer's instructions for Dynabead use. After 5 days, T_reg_ were washed with PBS and stained for 30 min at 37 °C with CellTracker Violet BMQC dye (ThermoFisher, Cat No. C10094, lot: 2451243). For staining, cells were washed with sterile PBS and resuspended at a concentration of 10^6^ cells mL^−1^ in a solution of PBS supplemented with CellTracker Violet BMQC dye, prepared by adding 2 µL of stock dye per 1 mL of PBS. Cells were then washed twice to remove residual dye and injected into a single ankle of either healthy BALB/cJ or SKG mice with established arthritis at 5 × 10^5^ T_reg_ suspended in 20 µL of PBS per mouse. Mice were sacrificed 3 days post‐injection and the spleens, draining lymph nodes (inguinal and popliteal), contralateral lymph nodes (inguinal and popliteal), injected ankle, and uninjected ankle were all harvested and analyzed using flow cytometry. Mice that did not receive any injection were used as a negative control for analysis.

### Fate‐Mapping Assays

A previously reported inducible T_reg_ fate‐mapping SKG mice (B6.SKG.H2d/d FoxP3^eGFP‐ERT2‐Cre+/+^ tdTomato^fl/fl^)^[^ ^41^ ^]^ was used. In these mice, FoxP3^eGFP+^ T_reg_ carry an inducible Cre‐ERT2 fusion protein that can be activated by administration of tamoxifen, allowing for generation of T_reg_‐specific fate mapping. To induce Cre expression, 8–12 weeks old male and female inducible T_reg_ fate‐mapping mice were administered 100 µL tamoxifen (T5648, MilliporeSigma, 20 mg mL^−1^ dissolved in corn oil) via oral gavage for 5 consecutive days. 1 week after the last treatment, mice were injected intraperitoneally with 20 mg of mannan dissolved in 200 µL of sterile PBS. 14 days after mannan injection, littermate pairs of mice were treated via IA injection of PLGA‐ATRA MP or PLGA‐Blank MP. 11 days following treatment, mice were sacrificed, and spleens, ankles, and ankle‐draining lymph nodes (popliteal and inguinal) were harvested and analyzed for expression of FoxP3, tdTomato, and IL‐17A.

### Micro‐CT Analysis

Mouse ankles were placed in 4% paraformaldehyde (PFA) for 48 h for fixation. After fixation, samples were transferred to 70% ethanol. Treatment‐blinded scanning and analysis was performed at the University of Gothenburg. Before scanning, bones were transferred to PBS for 24 h. Scanning was performed on a Skyscan1176 Micro‐CT (Bruker) with a voxel size of 9 µm, at 55 kV/467 mA, with a 0.2 mm aluminum filter. Exposure time was 880 ms. The X‐ray projections were obtained at 0.4° intervals with a scanning angular rotation of 180° and a combination of four average frames. The projection images were reconstructed into 3D images using Nrecon software (version 1.6.9.8, Bruker) and aligned for further analysis in DataViewer (version 1.5.0.9, Bruker). Data were processed using CT‐Analyzer software (version 1.14.4.1 Bruker), and images were generated using CTVox software (version 2.7, Bruker). Bone erosion was quantified as previously described.^[^ ^97^ ^]^

### Histological Processing

After sacrifice, mouse hind limbs were excised below the knee joint. Histology was from mice represented in clinical scoring in Figure 4 and Figure S7 in the Supporting Information. Muscle and skin were removed to the degree possible without damaging internal structures, and the limbs were fixed in 4% PFA for 48 h. The fixed limbs were then transferred to a 70% ethanol solution. Samples were then sent to the Tissues Technology Shared Resource Core at Moores Cancer Center (histology in Figure 5 and Figure S9 in the Supporting Information, except bolus ATRA), the University of Gothenburg (histology in Figure 9), or Inotiv (bolus ATRA histology in Figure S9, Supporting Information), where they were decalcified and embedded in paraffin. Paraffin embedded limbs were sectioned to an appropriate depth according to SMASH guidelines and stained with either H&E or safranin‐O using standard tissue processing techniques. Stained slides were digitized using an Aperio AT2 Automated Digital Whole Slide Scanner or a Zeiss Axioscan 7 Slide Scanner.

### Histomorphometry Analysis

To quantify immune cell infiltration in histological sections, H&E sections were loaded into the QuPath software (Figure S4c, Supporting Information). Representative histological sections were used to train the software using built‐in classification tools to broadly classify immune cells, muscle and tendon, and bone. Once trained, the software was then used to detect and classify types in sections from the metatarsals to part way up the tibia. Skin was excluded from the analysis to prevent misidentification of dermal cells as immune cells. For proteoglycan loss scoring and bone erosion scoring, SMASH guidelines were followed (30). Briefly, histological sections were examined and proteoglycan loss was scored as follows: 0: healthy intact cartilage consisting of fully stained cartilage layer with a smooth surface; 1: mild loss of staining in ≈1/3 of the superficial cartilage zone, still predominantly red when stained with Safranin O; 2: moderate loss of Safranin O staining in up to 2/3 of the superficial cartilage zone; 3: complete loss of Safranin O staining in the superficial cartilage zone. Bone erosion was scored as follows: 0: healthy, intact bone surface; 1: small, superficial bone erosion at the outer surface of the bone, no breakage into marrow; 2: enhanced local bone erosions into subchondral space, partial or complete penetration of cortical bone; 3: massive enlarged subchondral bone erosion, extended synovial pannus invasion causing near‐complete breakthrough of cortical bone to the marrow. Scoring was performed by two independent treatment‐blinded operators.

### Flow Cytometry Analysis

Anti‐mouse antibodies against IFN‐*γ* (clone: XMG1.2, lot: 2289505), IL‐4 (clone: BVD6‐24G2, lot: 2284161), IL‐17A (clone: eBio17B7, lot: 2142931), ROR*γ*t (clone: B2D, lot: 2304447), FoxP3 (clone: FJK‐16s, lot: 2199652) were purchased from Invitrogen. Anti‐mouse antibodies against CD8a (clone: 53‐6.7, lot: B313471) were purchased from Biolegend. Anti‐mouse antibodies against CD4 (cat: 1540‐26, lot: F2212‐T406C) were purchased from Southern Biotech. Anti‐human antibodies against FoxP3 (clone: 236A/E7, lot: 4336544) and ROR*γ*t (clone: AFKJS‐9, lot: 2123761) were purchased from Invitrogen. Anti‐human antibodies against IL‐17A (clone: BL168, lot: B252098), CD45 (clone: HI30, lot: B256106), and CD4 (clone: SK3, lot: B256770) were purchased from Biolegend. All cells were gated based on forward and side scatter characteristics to limit debris, including dead cells. The Zombie Aqua Fixable Viability Kit (Biolegend, lot: B333785) stain was used to separate live and dead cells. Antibodies were diluted according to the manufacturer's suggestions. Gates were drawn based on fluorescence‐minus‐one controls, and the frequencies of positively stained cells for each marker were recorded. Intracellular/intranuclear stains were performed by first staining for surface markers according to manufacturer's protocols, then fixing and permeabilizing cells using the FoxP3 Fixation/Permeabilization Buffer Set (Invitrogen, 00‐5523‐00, lots: 2333698, 2220750, 2203535). To quantify immune cell subsets in mouse ankles, ankles were harvested after sacrificing mice, skin was removed, and ankles were harvested and incubated at 37 °C in a solution of complete RPMI, 0.1 mg mL^−1^ Type VIII collagenase (Millipore‐Sigma, C2139‐100MG, lot: 0000090030), and 0.01 mg mL^−1^ DNAase (Millipore‐Sigma, 10104159001, lot: 60852700) for 60 min with intermittent gentle shaking. The supernatant was filtered through a 70 µm cell strainer and tissue was removed from the ankles and pressed through the screen and subsequently simulated with PMA/ionomycin (Biolegend, 00‐4970‐93, lot: 2430454) and brefeldin A (Biolegend, 00‐4506‐51, lot: 2229153) for 5 h, after which cells were stained. To quantify immune cell subsets in the spleen, spleens were mashed and filtered through 70 µm cell strainers using complete RPMI. Red blood cells were then lysed for 5 min, after which samples were washed with complete RPMI and stimulated with PMA/ionomycin and brefeldin A for 5 h. To quantify immune cells in lymph nodes, lymph nodes were mashed and filtered through 70 µm cell strainers using complete RPMI, and were concentrated via centrifugation, and subsequently stimulated with PMA/ionomycin and brefeldin A for 5 h. Cell counts and frequencies were calculated using flow cytometry. A portion of the spleen and lymph nodes were stained, and all cells from processed ankles were stained, unless otherwise noted. Flow cytometry was performed using an Attune NxT Acoustic Focusing cytometer analyzer (A24858) and data analyzed using FlowJo software.

For tetramer staining of C57BL/6J OVA reactive T cells, cells were incubated with the class II MHC IA‐(b) OVA tetramer QAVHAAHAEIN (OVA_325‐335_, NIH Tetramer Core Facility, Task Order 57395) for 1 h at 37 °C, after which cells were stimulated with PMA/ionomycin and brefeldin A without washing out tetramer for 4 h. Cells were subsequently washed and stained, fixed, and permeabilized according to manufacturer's instructions for the FoxP3 Fixation/Permeabilization Buffer Set (Invitrogen 00‐5523‐00, lot: 2333698).

### Ovalbumin Immunization Assays

In SKG mice, arthritis was induced via mannan injection as previously described. Arthritis treatments were administered IA on day 14, and mice were vaccinated on day 17 after with two 50 µL of an ovalbumin/complete Freund's adjuvant emulsion at two distinct sites subcutaneously on the back. Mice were bled 10 days after priming, and subsequently received booster immunizations of a single 100 µL injection of an ovalbumin/incomplete Freund's adjuvant subcutaneously. Mice were then bled and sacrificed 11 days after the booster injection.

In C57BL/6J mice, treatments were administered on day 0. A single IA dose of PLGA‐ATRA MP was administered IA. Intraperitoneal ATRA was administered daily for the duration of the experiment at 0.5 mg kg^−1^. On day 3, mice received a priming shot of 100 µL of an ovalbumin/complete Freund's adjuvant emulsion at one site on the back, administered subcutaneously. Mice were bled 13 days after priming, and subsequently received booster immunizations of a single 100 µL injection of an ovalbumin/incomplete Freund's adjuvant subcutaneously. Mice were then bled and sacrificed 11 days after the booster injection and stained as detailed in the flow cytometry section.

### Anti‐OVA Antibody Titer Analysis

Blood collected from the ovalbumin immunization assays were thawed and diluted in PBS at 1:3 peripheral blood to PBS ratios. Sera was extracted by spinning down at 800 *g* for 8 min and extracting the supernatant. Sera were then analyzed for IgG1 and IgG2a antibodies against ovalbumin using enzyme‐linked immunosorbent assay (ELISA, Biolegend 406603, lot: B270353 and Biolegend 407104, lot: B268019, respectively). High affinity plates were coated using OVA (Invivogen vac‐stova, lot: 5823‐43‐01) and anti‐OVA titers were defined as the highest serum dilution factor at which the ELISA optical density reading was ≥ 0.3.

### Statistical Analysis

Sample sizes for animal studies were based on prior work. The Biostatistics Unit of the Clinical and Translational Research Institute at UC San Diego was consulted on statistical analysis. Data outside three SD from the mean were excluded. Data were presented as either mean ± SD or mean ± SEM, as indicated for each experiment in figure legends. Results were analyzed where indicated using unpaired Student's one‐tailed *t*‐test, paired and unpaired Student's two‐tailed *t*‐test, one‐way analysis of variance (ANOVA) with post hoc multiple comparison test, repeated measures two‐way ANOVA with post hoc multiple comparison test (for clinical time course data with *n* < 5 per group), linear mixed effects model (for clinical time course data with *n* ≥ 5 per group), Mann–Whitney rank test, Wilcoxon match‐paired signed rank, and Kruskal–Wallis with post hoc multiple comparison test and identified for each individual experiment in the figure legends. *p*‐values are reported in figures. Data were analyzed using Graphpad Prism software for all except the linear mixed effects model analysis, which was analyzed in SPSS v28.

## Conflict of Interest

D.A.M., N.B., and N.J.S. are co‐inventors on a patent application related to the work described in the manuscript. N.J.S. is a founder of Tekhona Inc. and has an equity interest in the company. The terms of this arrangement have been reviewed and approved by the University of California, San Diego in accordance with its conflict of interest policies.

## Author Contributions

Conceptualization: D.A.M., N.B., N.J.S. Methodology: D.M., M.N.D.S., N.B., N.J.S. Investigation: D.A.M., M.D.K., W.T.J., N.C.D., A.N., M.Z. Validation: D.A.M., M.D.K., W.T.J., M.Z. Formal analysis: D.A.M., M.Z., M.Y., E.B.P., A.N. Data curation: D.A.M. Software: D.A.M. Visualization: D.A.M., A.N., M.Y., N.B., N.J.S. Funding acquisition: D.A.M., M.D.K., N.B., M.N.D.S., N.J.S. Project administration: N.B., N.J.S. Supervision: N.B., W.W., M.N.D.S., N.J.S. Resources: N.B., W.W., M.N.D.S., N.J.S. Writing—original draft: D.A.M., N.J.S. Writing–review & editing: D.A.M., M.D.K., M.Y., A.N., M.Z., M.N.D.S., N.B., N.J.S. All authors reviewed the data and analysis, provided input on the manuscript, and approved the submission.

## Supporting information

Supporting Information

## Data Availability

The data that support the findings of this study are available from the corresponding author upon reasonable request. Sequencing data in this publication have been deposited in the NCBI's Sequence Read Archive (SRA) database. ATAC‐Seq data are accessible through BioProject accession number PRJNA915588. CUT‐and‐TAG and RRBS data are accessible through BioProject accession number PRJNA922163.
