# Supplementary material for: Immunomodulatory Microparticles Epigenetically Modulate T Cells and Systemically Ameliorate Autoimmune Arthritis
Source: Adv Sci (Weinh). 2023 Mar 8;10(11):2202720. doi: 10.1002/advs.202202720 (PMC10104670; doi:10.1002/advs.202202720)
Supplement: Supplementary file 1 — Supporting Information [file ADVS-10-2202720-s001.pdf]

ADVANCED SCIENCE
Open Access

# Supporting Information

for *Adv. Sci.*, DOI 10.1002/advs.202202720

Immunomodulatory Microparticles Epigenetically Modulate T Cells and Systemically Ameliorate Autoimmune Arthritis

*David A. McBride, Matthew D. Kerr, Wade T. Johnson, Anders Nguyen, Martina Zoccheddu, Mina Yao, Edward B. Prideaux, Nicholas C. Dorn, Wei Wang, Mattias N.D. Svensson, Nunzio Bottini* and Nisarg J. Shah**

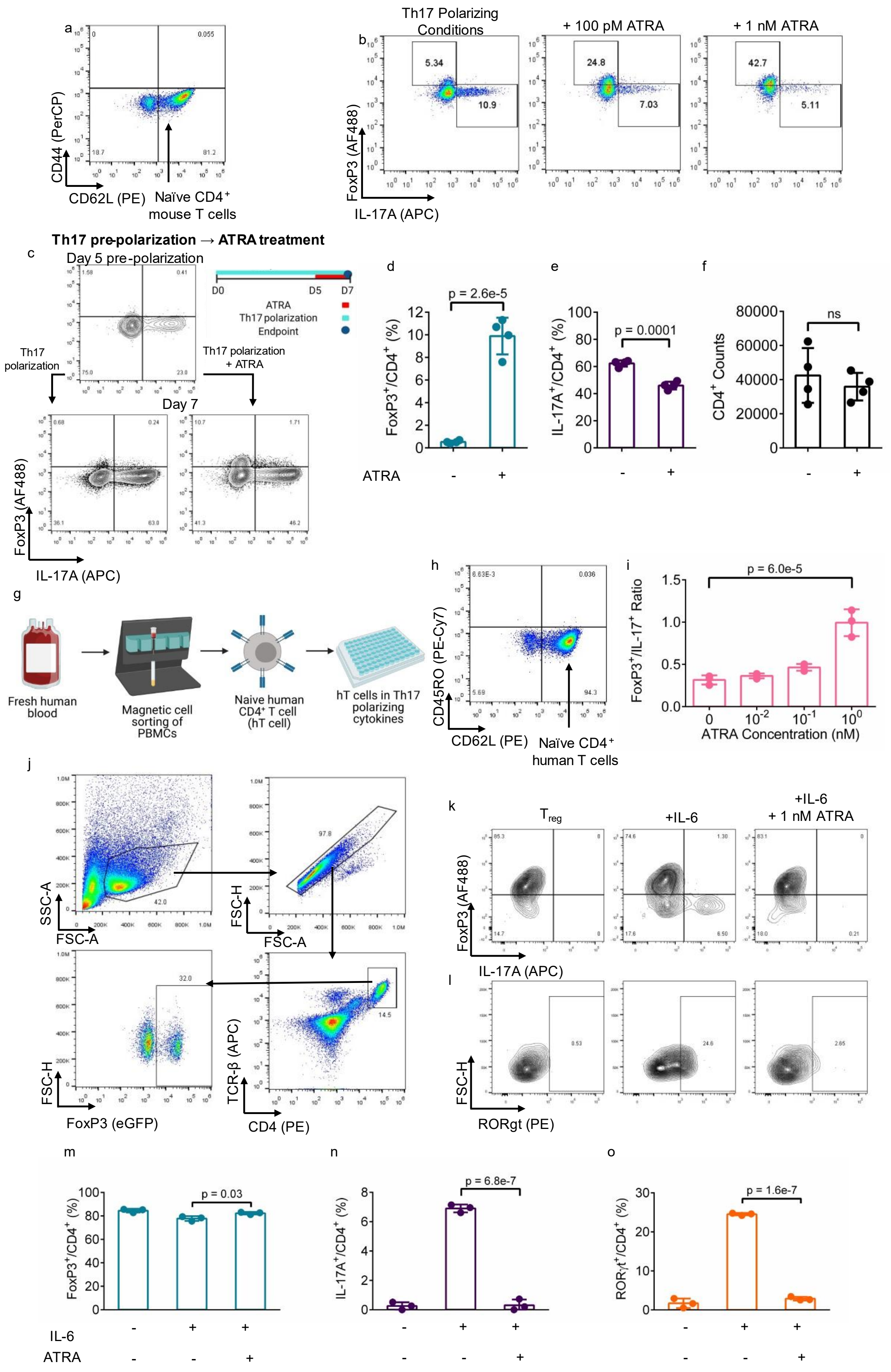
a
CD44 (PerCP)
CD62L (PE)
Naïve CD4+ mouse T cells
0.055
18.7
81.2
b
Th17 Polarizing Conditions
+ 100 pM ATRA
+ 1 nM ATRA
5.34
10.9
24.8
7.03
42.7
5.11
FoxP3 (AF488)
IL-17A (APC)
Th17 pre-polarization → ATRA treatment
c
Day 5 pre-polarization
D0
D5
D7
ATRA
Th17 polarization
Endpoint
Th17 polarization
Th17 polarization + ATRA
Day 7
FoxP3 (AF488)
IL-17A (APC)
d
p = 2.6e-5
FoxP3+/CD4+ (%)
ATRA
e
p = 0.0001
IL-17A+/CD4+ (%)
f
ns
CD4+ Counts
g
Fresh human blood
Magnetic cell sorting of PBMCs
Naive human CD4+ T cell (hT cell)
hT cells in Th17 polarizing cytokines
h
CD45RO (PE-Cy7)
CD62L (PE)
Naïve CD4+ human T cells
0.036
94.3
i
p = 6.0e-5
FoxP3+/IL-17+ Ratio
ATRA Concentration (nM)
j
SSC-A
FSC-A
FSC-H
FSC-A
FSC-H
FoxP3 (eGFP)
TCR-β (APC)
CD4 (PE)
42.0
97.8
32.0
14.5
k
Treg
+IL-6
+IL-6 + 1 nM ATRA
FoxP3 (AF488)
IL-17A (APC)
l
FSC-H
RORgt (PE)
m
p = 0.03
FoxP3+/CD4+ (%)
IL-6
ATRA
n
p = 6.8e-7
IL-17A+/CD4+ (%)
o
p = 1.6e-7
RORγt+/CD4+ (%)

**Supplementary Figure 1| Extended characterization of ATRA-mediated immunophenotypic modulation of T cells.** (a) Representative flow cytometry-based characterization of naïve $CD4^+$ SKG T cells post-isolation. (b) Representative flow cytometry-based characterization to assess the concentration dependent effect of ATRA on Th17 and $T_{reg}$ differentiation from naïve $CD4^+$ SKG T cells. (c) Timeline and representative flow cytometry of five days of pre-polarization in Th17 polarization conditions and day seven with or without the addition of 1 nM ATRA on day five. (d-f) Quantification of (d) FoxP3 expression, (e) IL-17 expression, (f) and total $CD4^+$ T cell counts from day seven after post-polarization, with or without ATRA added on day five. (g) Experimental schematic for assessing the effect of ATRA on Th17 and $T_{reg}$ differentiation from naïve $CD4^+$ human T (hT) cells. (h) Representative flow cytometry-based characterization of human naïve $CD4^+$ human T cells post-isolation. (i) Ratio of $FoxP3^+$ to IL-$17A^+$ human T cells at different concentrations of ATRA between 0-1 nM. (j) Representative flow cytometry gating for sorting of $TCR\beta^+CD4^+FoxP3eGFP^+$ $T_{reg}$. (k,l) Representative flow cytometry plots for (k) FoxP3, IL-17A, and (l) RORγt expression in $T_{reg}$ after a destabilization assay. (m-o) Quantification of (m) FoxP3 expression, (n) IL-17A expression and (o) RORγt expression in $T_{reg}$ following an IL-6 mediated destabilization assay with or without 1 nM ATRA, and compared with $T_{reg}$ stimulated without IL-6. Plots in (a), (b), (c), (h), (j)-(l), are representative flow cytometry plots of at least three experiments with at least three technical replicates each. Data in (d)-(f) are representative of the mean ± SD of four technical replicates. Data in (i) are representative of the mean ± SD of three technical replicates for a single donor. Data in (m)-(o) are representative of the mean ± SD of three technical replicates. Statistical analysis in (d)-(f) was performed using a Student's unpaired t-test. Statistical analysis in (i), (m)-(o) was performed using one-way ANOVA with post hoc Tukey test. Schematic in (c), (g) was composed in BioRender.

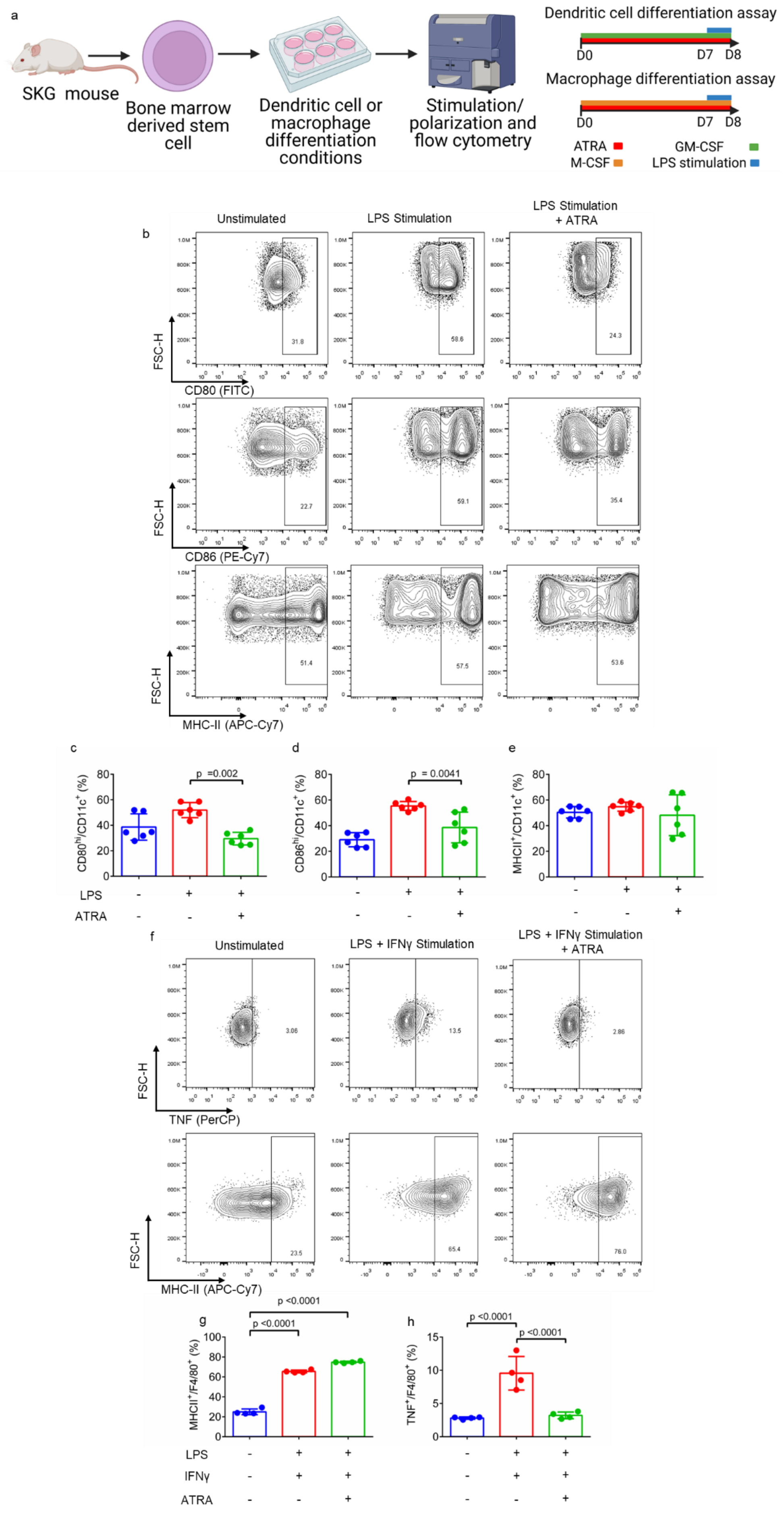
a
SKG mouse
Bone marrow derived stem cell
Dendritic cell or macrophage differentiation conditions
Stimulation/ polarization and flow cytometry
Dendritic cell differentiation assay
D0 D7 D8
Macrophage differentiation assay
D0 D7 D8
ATRA
M-CSF
GM-CSF
LPS stimulation
b
Unstimulated
LPS Stimulation
LPS Stimulation + ATRA
FSC-H
CD80 (FITC)
31.8
58.6
24.3
CD86 (PE-Cy7)
22.7
59.1
35.4
MHC-II (APC-Cy7)
51.4
57.5
53.6
c
CD80hi/CD11c+ (%)
p =0.002
d
CD86hi/CD11c+ (%)
p = 0.0041
e
MHCII+/CD11c+ (%)
LPS
ATRA
f
Unstimulated
LPS + IFNγ Stimulation
LPS + IFNγ Stimulation + ATRA
TNF (PerCP)
3.06
13.5
2.86
MHC-II (APC-Cy7)
23.5
65.4
76.0
g
MHCII+/F4/80+ (%)
p <0.0001
p <0.0001
h
TNF+/F4/80+ (%)
p <0.0001
p <0.0001
LPS
IFNγ
ATRA

**Supplementary Figure 2| Extended characterization of ATRA-mediated immunophenotypic modulation of dendritic cells (DCs) and macrophages.** (a) Experimental schematic and timeline for testing ATRA-mediated modulation of DCs and macrophages. (b) Representative flow cytometry of activation markers in unstimulated, stimulated, and stimulated + ATRA treated DCs. (c) $CD80^+CD11c^+$ dendritic cells, (d) $CD86^+CD11c^+$ dendritic cells  dendritic cells and (e) $MHCII^+CD11c^+$ dendritic cells. (f) Representative flow cytometry of activation markers in unstimulated, stimulated, and stimulated + ATRA treated $F4/80^+$ macrophages. (g) $MHCII^+F4/80^+$ macrophages and (h) $TNF^+F4/80^+$ macrophages. Data in (c)-(e), (g), (h) are mean ± SD of technical replicates from a representative experiment. Experiments in (c)-(e), (g),(h) were performed twice. Statistical analyses in (c)-(e), (g), (h) were performed using one-way ANOVA with a post hoc Tukey multiple comparison test; schematic in (a) was composed in Biorender.

a

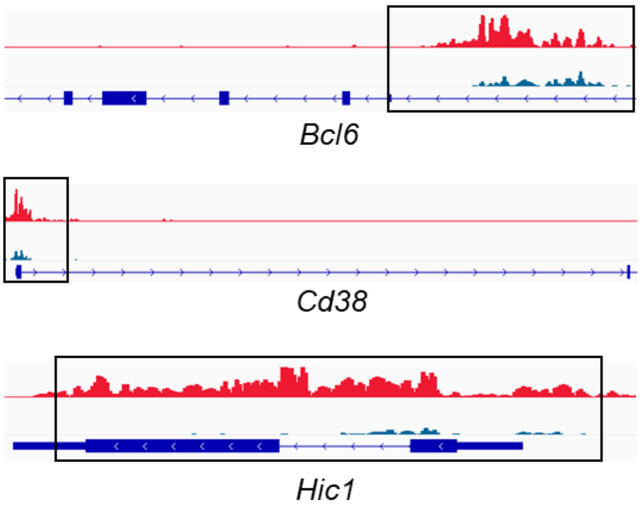


b

| List of Differentially Methylated Gene IDs |
|---|
| 1700044K03Rik |
| Tspear |
| Tspear |
| Prnd |
| 1700016K19Rik |
| Gm26578 |

**Supplementary Figure 3| Extended characterization of ATRA-mediated epigenetic changes in CD4+ SKG T cells.** (a) Visualization of H3K4me3 analysis at additional differentially methylated sites associated with $T_{reg}$/Th17. (b) List of differentially methylated loci between -ATRA and +ATRA treated CD4+ SKG T cells from RRBS analysis. Scales in (a): *Hic1* [0-44], *Bcl6* [0-24], *Cd38* [0-62].

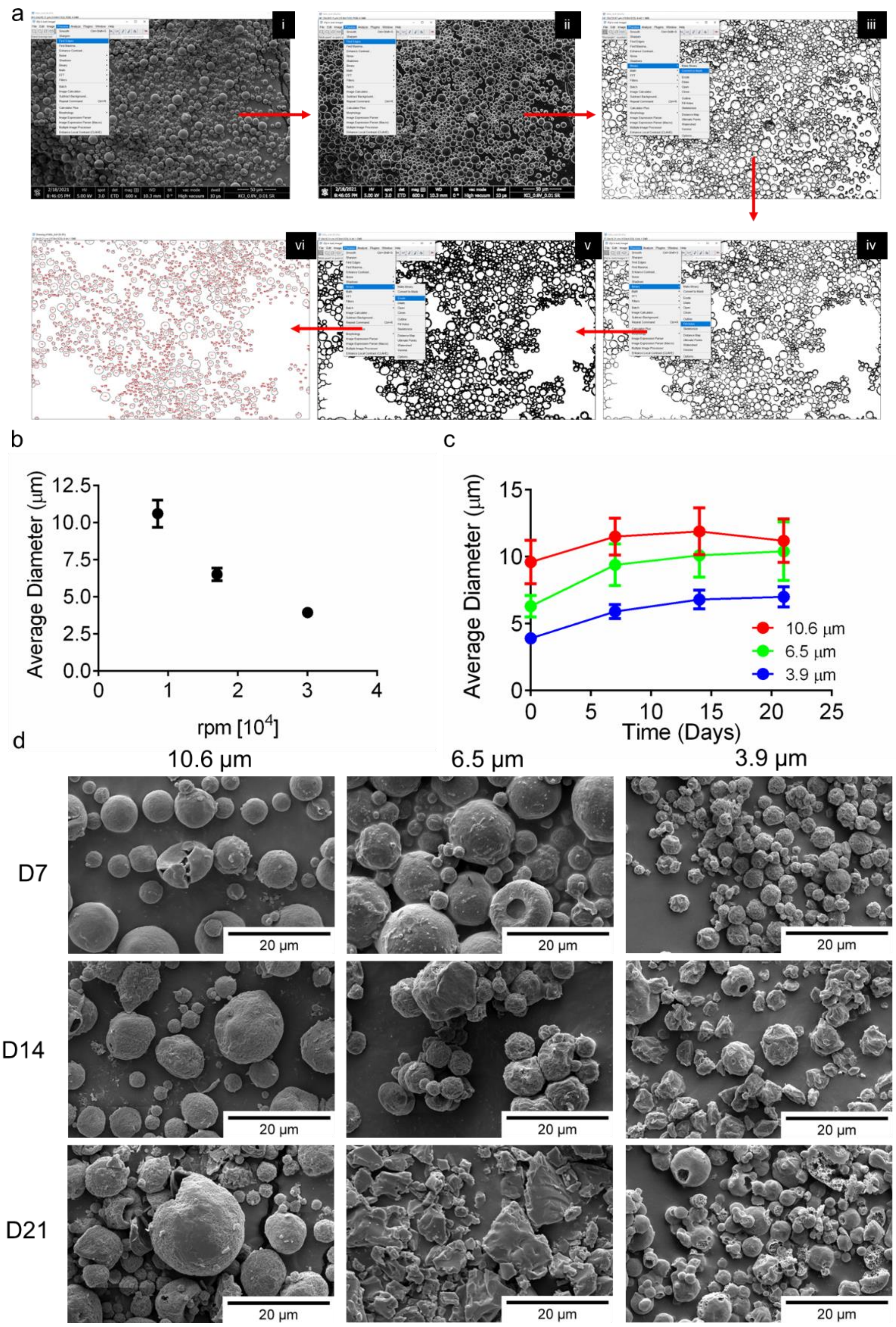


**Supplementary Figure 4| Extended characterization of particle size analysis.** (a) Scanning electron micrograph images were loaded into ImageJ (FIJI distribution) and analyzed in a six step process in which i) the scale of the image was set using the "Set Scale" function, ii) "Find Edges" was applied to find the microparticle edges, iii) image scale bars were cropped out and the image was converted to a black and white binary mask using the "Convert to Mask" function, iv) as much interior was accounted for as possible using the "Fill Holes" function

(Note: this loses some particles, but improves measurement accuracy as long as multiple images from the same sample are analyzed), v) edges were expanded using the “Erode” function to avoid detection of aggregates as a single particle and, vi) the area of the particles was analyzed using the “Analyze Particles” function. A subsequent manual check was performed to ensure that no background was incorrectly counted as particles, and particle areas were converted to diameters. The erode function slightly reduces the calculated particle diameters based on the magnification of the image, which was accounted for in final size distribution analyses. (b) Particle size as a function of homogenization speed. (c) Quantification of particle sizes over the duration of four weeks of incubation in release buffer. (d) Representative SEM images depicting degradation of PLGA-ATRA MP over four weeks of incubation in release buffer. Data in (b) represents mean ± SD of average particle diameter from three batches; data in (c) represents the mean ± SD of average particle diameter from three representative images.

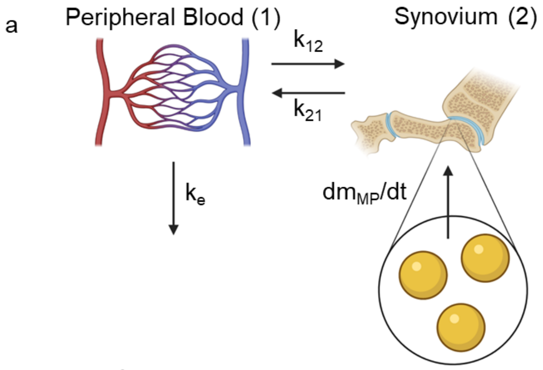


b

$$(1)\ \frac{dC_1}{dt} = -k_{12}C_1 + k_{21}C_2 z\ - k_e C_1$$

$$(2)\ \frac{dC_2}{dt} = \frac{k_{12}C_1}{z} - k_{21}C_2 + \frac{dm_{MP}(t)}{dt}\frac{1}{V_2}$$

| Parameter | $k_{12}$ | $k_{21}$ | $k_e$ | $V_2$ | z |
|---|---|---|---|---|---|
| Value | 0.2 $h^{-1}$ | 0.2 $h^{-1}$ | 0.84 $h^{-1}$ | 20 μL | 0.0013 |

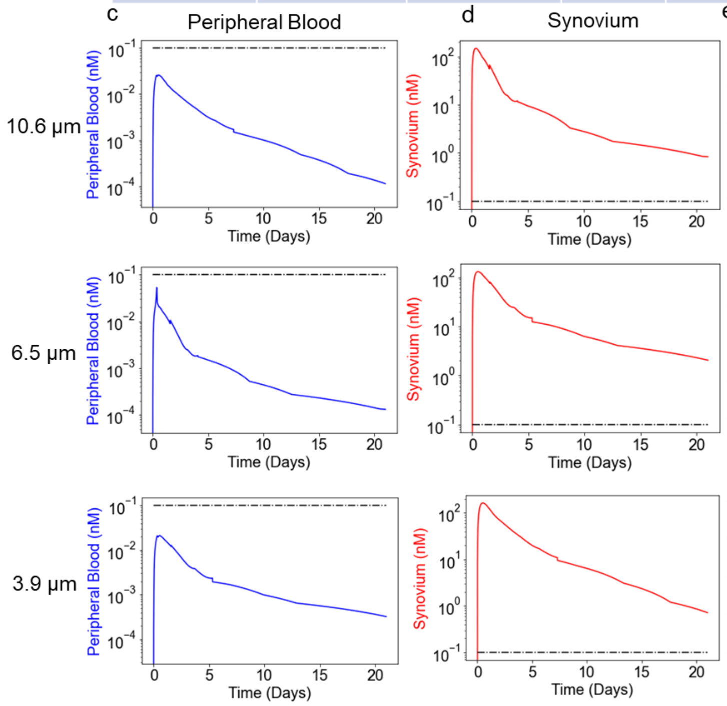


e

Key Model Outputs

| $C_{max,1}$ | $t_{max,1}$ |
|---|---|
| 16 pM | 11.1 hr |
| $C_{max,2}$ | $t_{max,2}$ |
| 130 nM | 10.3 hr |

| $C_{max,1}$ | $t_{max,1}$ |
|---|---|
| 40 pM | 7.3 hr |
| $C_{max,2}$ | $t_{max,2}$ |
| 146 nM | 8.1 hr |

| $C_{max,1}$ | $t_{max,1}$ |
|---|---|
| 20 pM | 10.3 hr |
| $C_{max,2}$ | $t_{max,2}$ |
| 162 nM | 9.9 hr |

**Supplementary Figure 5| Pharmacokinetic modeling of PLGA-ATRA MP.** (a) Schematic of a two-compartment pharmacokinetic (PK) model for the release of ATRA from PLGA-ATRA MP, transfer of ATRA between the synovium and peripheral blood, and elimination of ATRA from the peripheral blood. (b) Governing differential equations for the model compartments and parameter values for first order model coefficients. (c) Model-computed concentration profiles in the peripheral blood based on in vitro release profiles of PLGA-ATRA MP. (d) PK-modeled concentration profiles of ATRA in the synovial fluid. (e) Maximum concentration ($C_{max}$) and the time at which it occurs ($t_{max}$) values. Data in (c)-(e) represent model

outputs using the parameters and the in vitro ATRA release profile from PLGA-ATRA MP. Schematic in (a) was composed in BioRender.

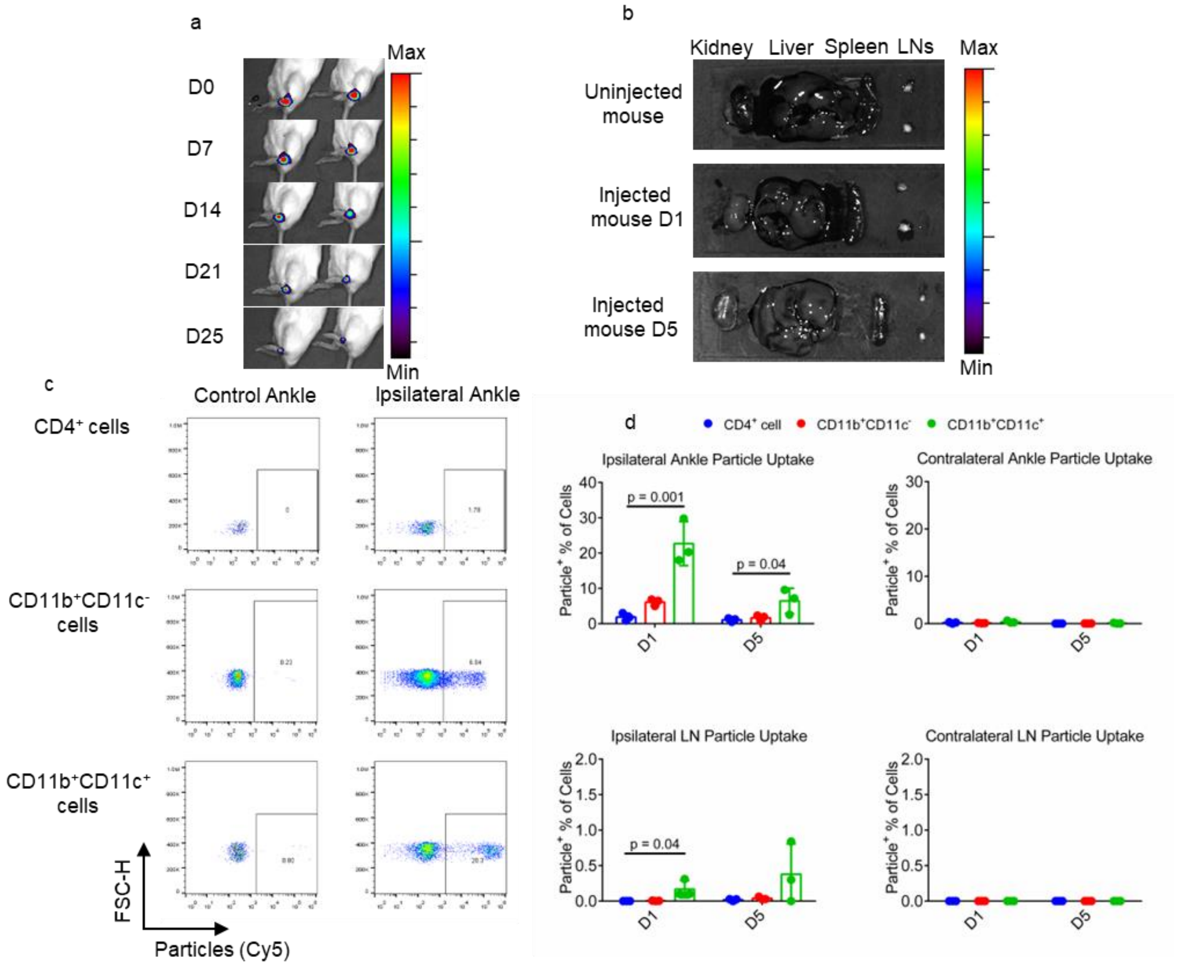


**Supplementary Figure 6| Analysis of PLGA MP retention and uptake in vivo.** (a) Representative IVIS images of data quantified in Fig. 4b showing fluorescence from IA injected Cy5-tagged PLGA-Blank MP. (b) Representative IVIS images depicting Cy5 signal in kidneys, livers, spleens, and popliteal lymph nodes (LNs) (c) Representative flow cytometry-based characterization of particle uptake by $CD4^+$, $CD11b^+CD11c^-$ and $CD11b^+CD11c^+$ cells isolated from ankle joints. (d) Quantification of particle uptake by various cell populations in the ipsilateral and contralateral ankles and the respective draining lymph nodes. Scales in (a) and (b) are different. Data in d represent the mean ± SD. Statistical analysis in (d) was performed using a one-way ANOVA with a post hoc Tukey multiple comparison test.

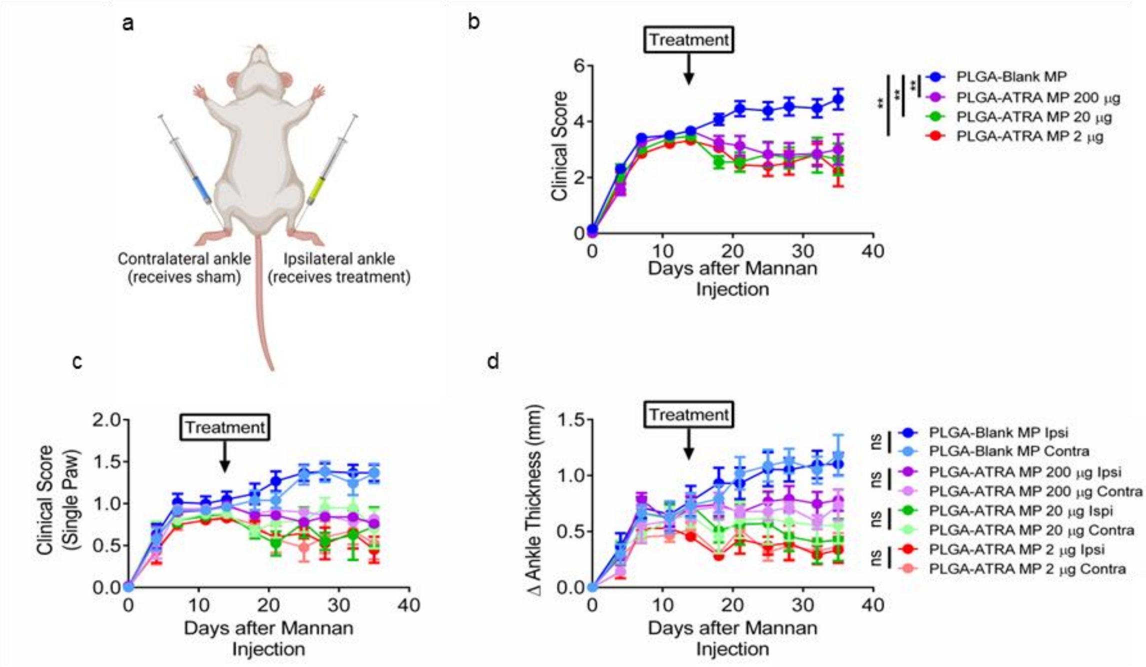


**Supplementary Figure 7| Extended characterization of PLGA-ATRA MP modulating autoimmune arthritis in SKG mice.** (a) Schematic depicting IA injections in the ipsilateral and contralateral ankles. (b) Aggregated and (c) disaggregated ipsilateral and contralateral ankle clinical scores and (d) disaggregated ankle thickness measurements in mice treated with either PLGA-Blank MP or PLGA-ATRA MP at 2, 20, or 200 µg doses. Schematic in **a** generated using Biorender. Data for groups in b-d, were powered as follows: PLGA-Blank MP (n = 8), PLGA-ATRA MP 200 µg (n = 6), PLGA-ATRA MP 20 µg (n= 4), PLGA-ATRA MP 2 µg (n=4). ** $p < 0.01$. Data in (b)-(d) represent mean ± SEM. Statistical analyses for (b)-(d) were performed using repeated measures two-way ANOVA with post hoc Tukey test. Differences between ipsilateral and contralateral scores in (c) and (d) were not significant; schematic in (a) was composed in Biorender.

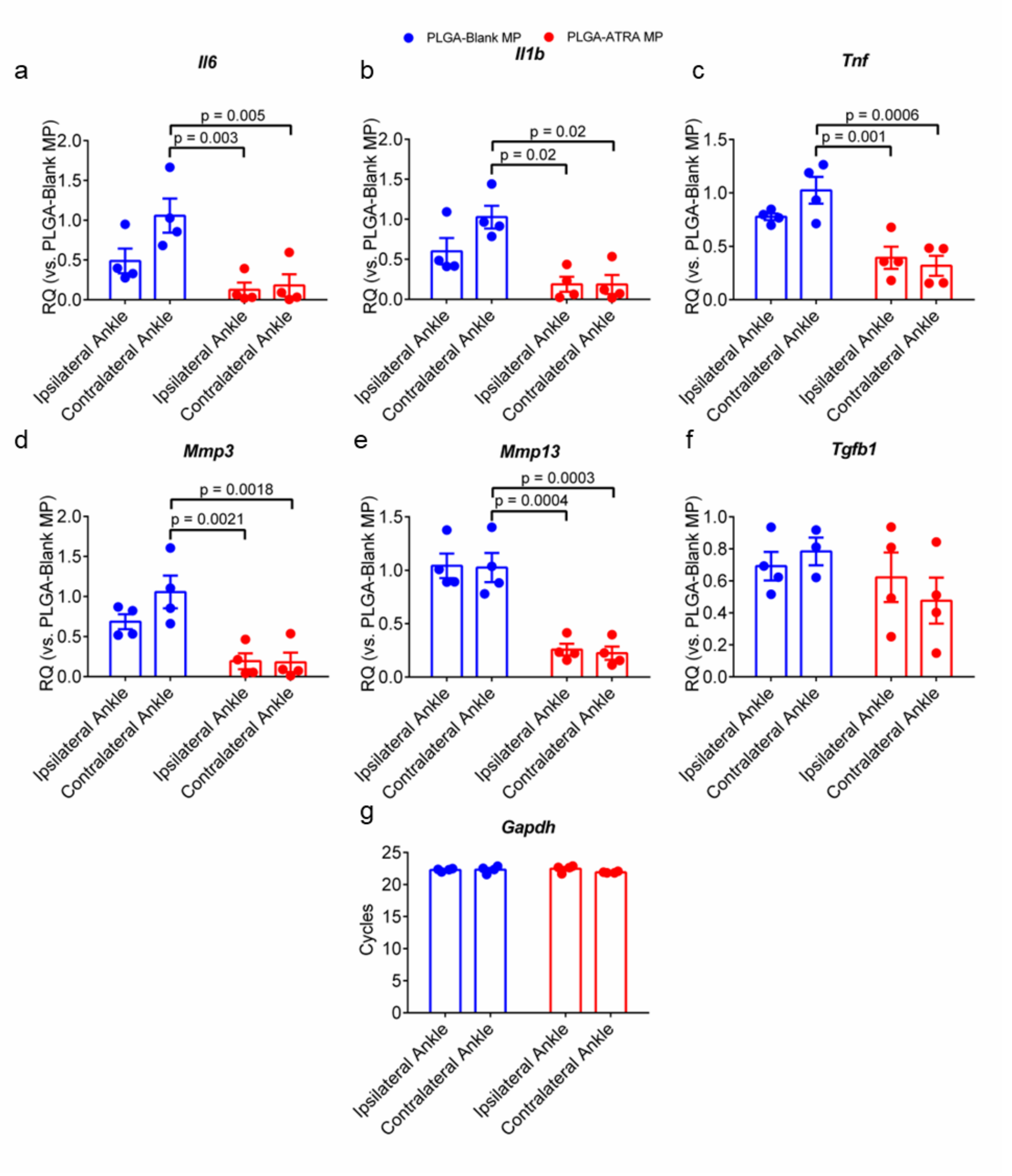


**Supplementary Figure 8| Extended characterization of PLGA-ATRA MP-mediated reduction in joint inflammation in ipsilateral and contralateral ankles.** (a-f) qPCR relative quantification of (a) *Il6*, (b) *Il1b*, (c) *Tnf* (d) *Mmp3* (e) *Mmp13* and (f) *Tgfb*. (g) *Gapdh* cycle counts from qPCR. Data in a-f represent the mean ± SD; data in (a)-(f) are normalized first to *Gapdh* from respective samples then normalized to mRNA expression in PLGA-Blank MP contralateral ankles before comparison between treatment groups. Statistical analysis performed using a one-way ANOVA with a post hoc Dunnett's multiple comparison test.

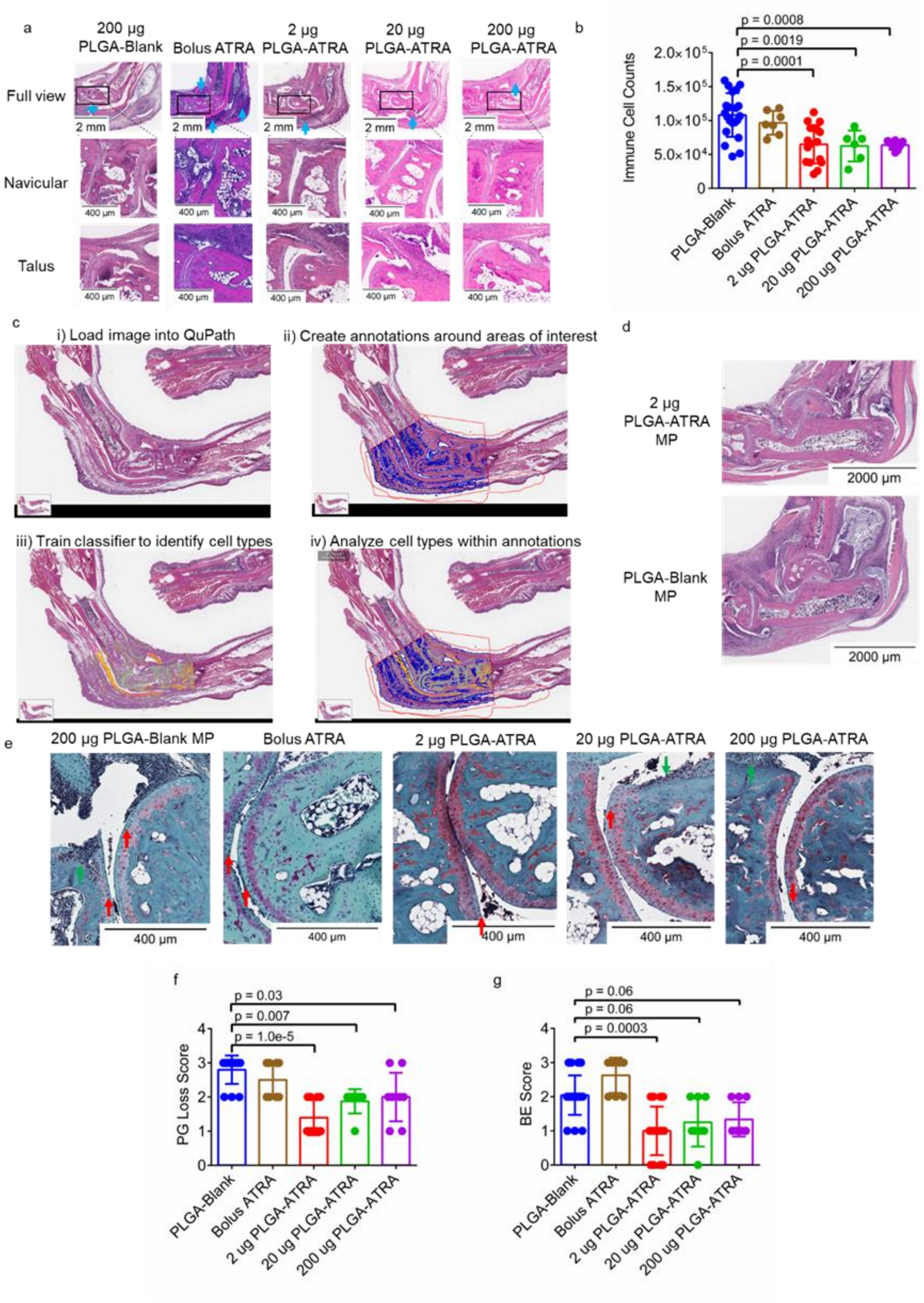


**Supplementary Figure 9| Extended characterization of IA PLGA-ATRA MP-mediated reduction in immune cell infiltration, cartilage damage and bone erosions in treated mice.** (a) Representative H&E-stained histological sections of ankles from arthritic SKG mice, treated as described in Fig. 4a with indicated treatments. Immune cells (blue arrow) are indicated and (b) quantified using (c) QuPath software-based histomorphometry analysis

following the depicted workflow. (d) Representative H&E sections of contralateral ankles from mice treated with either 2 µg PLGA-ATRA MP or PLGA-Blank MP in the ipsilateral ankle. (e) Representative safranin-O-stained histological sections with bone erosion (green arrows) and proteoglycan loss (red arrows) from SKG mice treated as described in Fig. 4a with indicated treatments. (f,g) Quantification of (f) proteoglycan (PG) loss and (g) bone erosion (BE) reported as aggregated scores from both ankles of mice treated as indicated. Data in (b), (f), (g) represent mean ± SD. Comparison groups are powered as follows: PLGA-Blank MP (n = 21 sections from 11 mice), bolus ATRA (n = 7 sections from 4 mice), 2 µg PLGA-ATRA MP (n = 16 sections from 9 mice), 20 µg PLGA-ATRA MP (n = 6 sections from 3 mice) and 200 µg PLGA-ATRA MP (n = 8 sections from 5 mice). Images for 2 µg PLGA-ATRA MP and PLGA-Blank MP in (a) and (e) are the same images as in Figure 5a, d respectively. Statistical analysis in (b) was performed using a one-way ANOVA with post-hoc Tukey test; statistical analysis in (f), (g) was performed using a Kruskal-Wallis test with post-hoc Dunn’s test. Histology sections included in (a) are from samples obtained in three separate experiments.

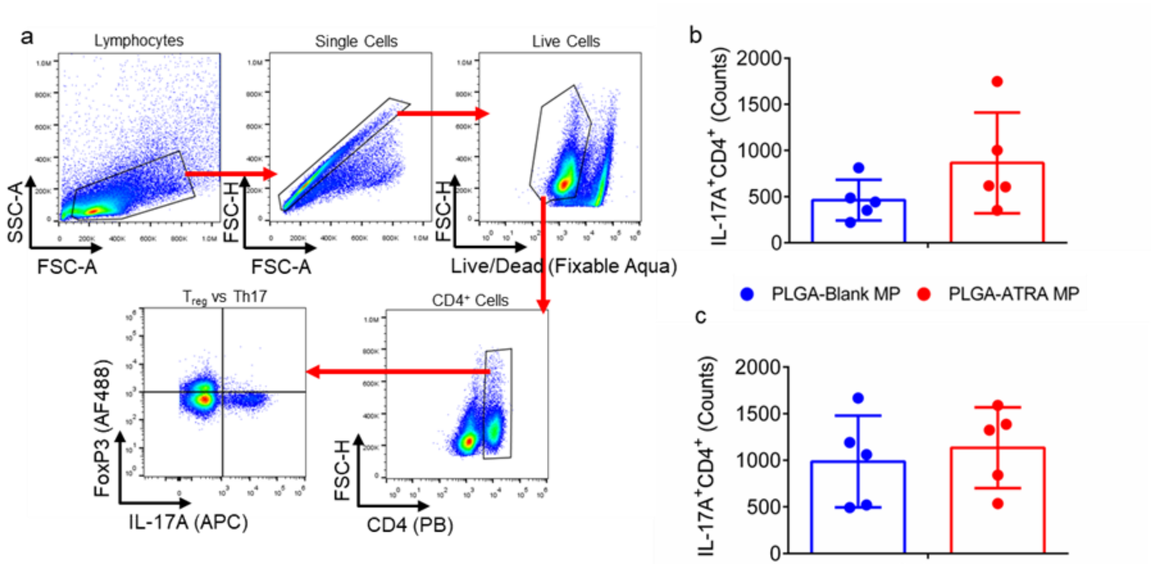


**Supplementary Figure 10| Extended characterization of ATRA-enhanced T$_{reg}$.** (a) Representative gating strategy for data in Figure 6d-g. (b, c) Quantification of IL-17A$^+$CD4$^+$ T cells in the spleens isolated from mice treated with PLGA-ATRA MP or PLGA-Blank MP on (b) day 17 or (c) day 25 after mannan injection. Data in b, c represent the means ± SD (n = 5/group). Statistical analysis in (b), (c) was performed using Student's unpaired t-test, differences were not significant.

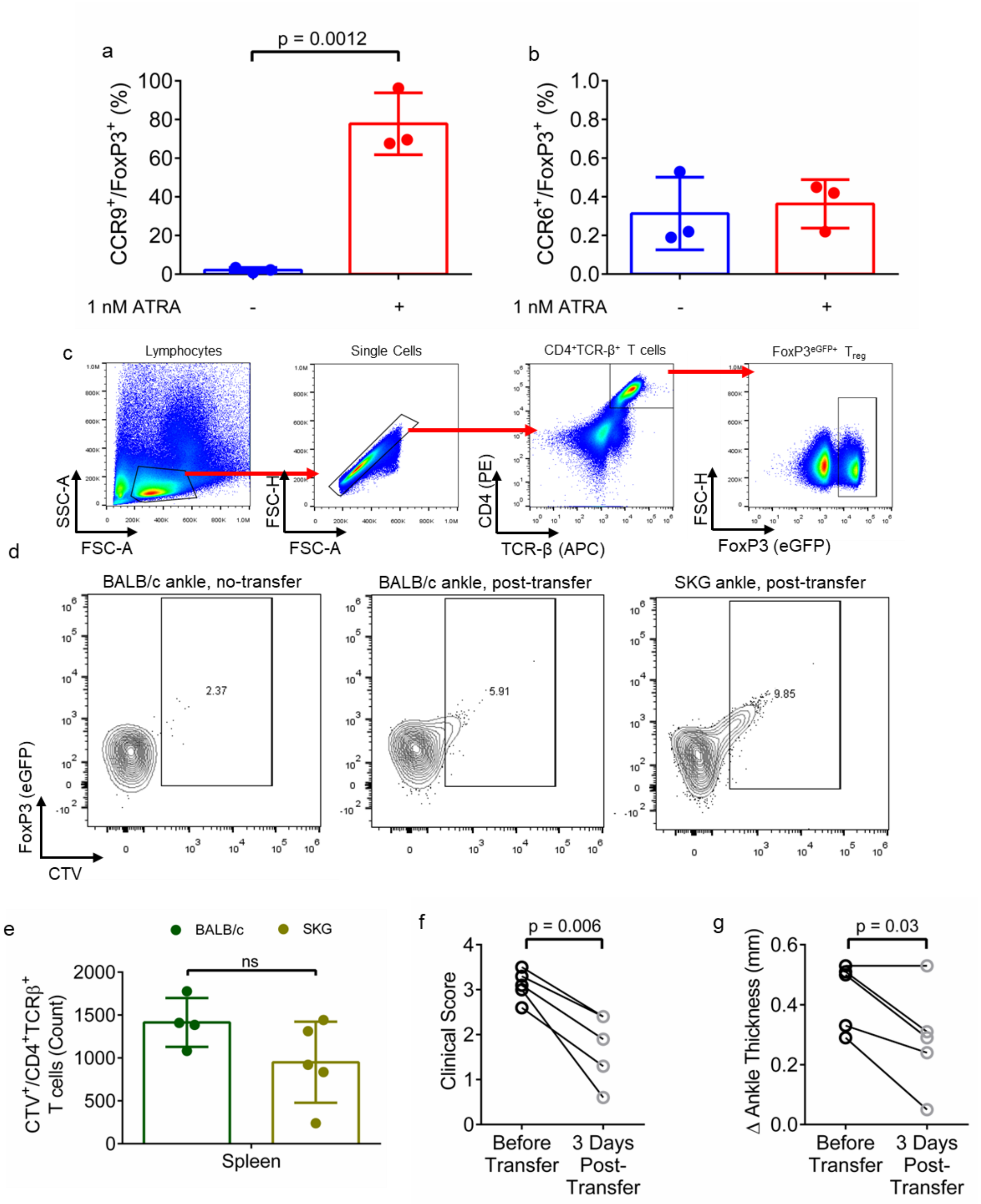


**Supplementary Figure 11| Extended characterization of the immunomodulatory effect of ATRA and PLGA-ATRA MP on $T_{reg}$.** (a, b) Quantification of (a) CCR9 expression and (b) CCR6 expression in ex vivo differentiated $T_{reg}$ cultured with or without 1 nM ATRA. (c) Representative flow cytometry gating strategy for sorting $T_{reg}$. (d) Representative flow cytometry plots of CellTracker Violet$^+$ (CTV$^+$) $T_{reg}$ in the ankles. (e) Quantification CTV$^+$CD4$^+$TCR-β$^+$ T cells in the spleen three days after IA injection. (f) Clinical scores of arthritic SKG mice prior to and three days after IA $T_{reg}$ transfer. (g) Change in ankle thickness

relative to ankle thickness before arthritis onset in the contralateral ankles of arthritic SKG mice prior to and three days after IA $T_{reg}$ transfer. Data in (a), (b), (e) represent the means ± SD (n = 5/group); data in (f), (g) represent clinical scores and change in ankle thicknesses of individual mice (n = 5) respectively prior to and three days after $T_{reg}$ transfer. Data in (a), (b) are technical replicates from a single experiment, which was performed twice. Statistical analyses in (a), (b), (e) were performed using an unpaired Student's two tailed t-test. Statistical analyses in (f) and (g) were performed using a paired Student's two tailed t-test.
